# Progesterone Receptor Expression Declines in the Guinea Pig Uterus during Functional Progesterone Withdrawal and in Response to Prostaglandins

**DOI:** 10.1371/journal.pone.0105253

**Published:** 2014-08-26

**Authors:** Toni N. Welsh, Jonathan J. Hirst, Hannah Palliser, Tamas Zakar

**Affiliations:** 1 Department of Obstetrics and Gynaecology, John Hunter Hospital, New Lambton Heights, New South Wales, Australia; 2 School of Biomedical Science and Pharmacy, University of Newcastle, Callaghan, New South Wales, Australia; 3 School of Medicine and Public Health, University of Newcastle, Callaghan, New South Wales, Australia; 4 Mothers and Babies Research Centre, New Lambton Heights, New South Wales, Australia; 5 Hunter Medical Research Institute, New Lambton Heights, New South Wales, Australia; Michigan State University, United States of America

## Abstract

Progesterone withdrawal is essential for parturition, but the mechanism of this pivotal hormonal change is unclear in women and other mammals that give birth without a pre-labor drop in maternal progesterone levels. One possibility suggested by uterine tissue analyses and cell culture models is that progesterone receptor levels change at term decreasing the progesterone responsiveness of the myometrium, which causes progesterone withdrawal at the functional level and results in estrogen dominance enhancing uterine contractility. In this investigation we have explored whether receptor mediated functional progesterone withdrawal occurs during late pregnancy and labor in vivo. We have also determined whether prostaglandins that induce labor cause functional progesterone withdrawal by altering myometrial progesterone receptor expression. Pregnant guinea pigs were used, since this animal loses progesterone responsiveness at term and gives birth in the presence of high maternal progesterone level similarly to primates. We found that progesterone receptor mRNA and protein A and B expression decreased in the guinea pig uterus during the last third of gestation and in labor. Prostaglandin administration reduced while prostaglandin synthesis inhibitor treatment increased progesterone receptor A protein abundance. Estrogen receptor-1 protein levels remained unchanged during late gestation, in labor and after prostaglandin or prostaglandin synthesis inhibitor administration. Steroid receptor levels were higher in the non-pregnant than in the pregnant uterine horns. We conclude that the decreasing expression of both progesterone receptors A and B is a physiological mechanism of functional progesterone withdrawal in the guinea pig during late pregnancy and in labor. Further, prostaglandins administered exogenously or produced endogenously stimulate labor in part by suppressing uterine progesterone receptor A expression, which may cause functional progesterone withdrawal, promote estrogen dominance and foster myometrial contractions.

## Introduction

The maintenance of pregnancy depends on adequate levels of the steroid hormone progesterone in the maternal circulation. In a number of mammalian species including the mouse, rat and sheep, progesterone levels fall and estrogen levels rise at the end of pregnancy, which is followed by the delivery of the fetus [1]. Administration of progesterone prolongs gestation and interventions that decrease progesterone levels before term stimulate the delivery of premature offspring with high rates of morbidity and mortality. In other mammals that include humans, non-human primates and the histricognath rodent guinea pig (Cavia porcellus), parturition occurs without a fall in progesterone and a rise in estrogen concentrations in the maternal plasma at the time of labor [2]–[4]. Progesterone administration does not prolong pregnancy beyond term in these species, but progesterone is critical for maintaining pregnancy earlier during gestation. Spontaneous preterm birth, which is a major health problem in humans, also occurs without a decline of progesterone concentration in the maternal blood.

The mechanism that determines gestational length and initiate parturition is unknown in mammals that give birth without the withdrawal of circulating maternal progesterone. One possibility suggested by the analysis of myometrial and cervical tissue samples from women and non-human primates is that the expression of the two nuclear progesterone receptor isoforms, PRA and PRB, changes at term leading to a decrease in the efficacy of progesterone to maintain uterine quiescence [5]–[8]. Diminished target tissue responsiveness is functionally equivalent to the withdrawal of circulating progesterone and may also result in increased myometrial contractility. Further support for this mechanism, often called functional progesterone withdrawal, was provided by studies showing that prostaglandins, which are powerful stimulants of parturition, alter progesterone receptor levels in cultured myometrial and decidua cells potentially decreasing progesterone responsiveness [9] [10]. There is no evidence, however, that this mechanism operates in vivo and the factor(s) eliciting functional progesterone withdrawal in term and preterm parturition are still undefined.

In the present investigation we have explored the mechanism of functional progesterone withdrawal by determining whether (i) a decline in progesterone receptor expression occurs in the uterus during normal pregnancy in preparation for birth and (ii) prostaglandins can cause functional progesterone withdrawal by altering myometrial progesterone receptor expression *in vivo*. In addition, we have measured myometrial estrogen receptor expression in late pregnancy and in response to prostaglandin to determine if the estrogen responsiveness of the uterus changes at the functional level to promote estrogen action in the absence of rising circulating estrogen concentrations before labor. Timed-pregnant guinea pigs were used in the experiments, since this animal gives birth without a fall of progesterone and a rise in estrogen concentrations in the maternal plasma at term labor [4]. Furthermore, prostaglandins induce birth and intrauterine prostaglandin production increases in late gestation and in labor in guinea pigs [11]–[13]. These hormonal conditions and responses closely resemble those observed in term pregnant women, which makes the guinea pig an informative animal model to study the mechanism of functional progesterone withdrawal in the late stages of gestation in vivo.

## Materials and Methods

### Animals and Tissues

Ethics statement: All procedures involving animals were approved by The University of Newcastle Animal Care and Ethics Committee (Approval number: 944 0607). Euthanasia was performed immediately after the procedures by exposure to CO_2_ without the use of anaesthetics, following AVMA guidelines[14]. Reporting of animal studies follows the ARRIVE guidelines as appropriate [15].

Timed-mated pregnant guinea pigs of outbred tricolour strain were obtained from the animal care facility of the John Hunter Hospital, Newcastle, NSW. Established pregnant animals were housed in individual cages in the proximity of other members of the colony, kept under a 12 h∶12 h dark-light cycle and fed ad libitum with standard guinea pig diet. Uteri were collected at the following stages of pregnancy as described before[13], [16]: 44–47 days (45d), 52–56 days, before the attachment of the visceral yolk sac to the endometrium (55dA), 54–56 days, following the attachment of the visceral yolk sac to the endometrium (55dB), 57–65 days, the first day of palpable pubic symphysis separation (62d), 62–66 days, the fifth day following pubic symphysis separation (67d) and during labor, following the delivery of at least one, but not all, pups (Labor). All groups comprised 8 randomly assigned guinea pigs, except for group Labor, which had 6 randomly assigned animals. The uteri were excised at approximately 5 mm above the cervix, dissected, and the fetuses and placentas were removed. The tissues were then frozen immediately in liquid nitrogen. In cases of unilateral pregnancies the non-pregnant uterine horn was cut off and frozen separately.

### Drug Treatments

Sulprostone (Cayman Chemical, Cat. 14765) was dissolved in sterile phosphate buffered saline (PBS), and 300 µL solution, containing 0.28±0.03 mg/kg (mean, SD) of the drug, was injected subcutaneously (s.c.) in 46d pregnant guinea pigs (n = 8). Control animals were injected with 300 µL sterile PBS (n = 8). Uteri were collected at 13–17 h after the injections. Six of the 8 Sulprostone treated animals remained intact and two aborted partially by the time of tissue collection.

The Piroxicam treatment protocol was described previously [13]. Briefly, Piroxicam (Sigma Cat. P0847-1KG) was dissolved in a mixture of canola oil:DMSO 3∶1, and 200 µL solution containing 5 mg/kg of the drug was injected s.c. once every day from 55d of pregnancy until the 5^th^ day following palpable pubic symphysis separation (60–63d of pregnancy, n = 8). Uteri were collected on the next day. Control animals (n = 7) were treated with 200 µL vehicle per day for the same period of time. This Piroxicam treatment regime significantly prolongs pregnancy in guinea pigs [13]. Animals were assigned randomly to the vehicle and drug treatment groups. The drugs caused no adverse effects as determined by the regular monitoring of animals.

### Immunoblotting

Frozen uterine tissue was pulverised in liquid nitrogen, and 0.2 g samples were homogenised with a Polytron-type homogeniser in 2 ml ice cold RIPA buffer (50 mM TrisHCl, pH 7.5; 150 mM NaCl; 1% v/v NP40, 0.5% sodium deoxycholate, 0.1% SDS) supplemented with Roche (Dee Why, NSW, Australia) cOmplete Mini Protease Inhibitor Cocktail (1 tablet/10 ml) and Roche PhosSTOP Phosphatase Inhibitor Cockail (1 tablet/10 ml). Protein concentration in the supernatants (obtained at 12,000 g, 10 min at 4C) was determined by the BCA assay (Pierce-Thermo Fisher Scientific, Scoresby, VIC, Australia).

Extracted proteins were separated by SDS-gel elecrophoresis using the NuPAGE gel system from Invitrogen (Life Technologies, Mulgrave, VIC, Australia). For PR immunoblots, aliquots of the supernatants were supplemented with NuPAGE sample buffer and reducing agent, heated at 70C for 10 min and loaded into wells of 10% Bis-Tris precast gels. One tissue- or cell (T47D or MCF-7) extract served as calibrator and was run on all gel slabs to correct for gel-to-gel variation. Magic Mark XP standard mixture (Invitrogen) was loaded in a separate lane to assess molecular weights based on relative mobility (M_r_). Proteins were separated using MOPS running buffer as per the manufacturer's instructions (Invitrogen). For ESR1 immunoblots, aliquots of supernatants containing 3 mg protein were adjusted to 1 ml with water, and precipitated by adding 500 µl of saturated ammonium sulphate on ice for 30 min (33% saturation). The precipitate was collected with centrifugation (10,000 g, 10 min 4 C) and dissolved in 100 or 200 µl water. Protein content was measured with the BCA procedure. The redissolved protein precipitates were processed for electrophoresis as above.

**Figure 1:**
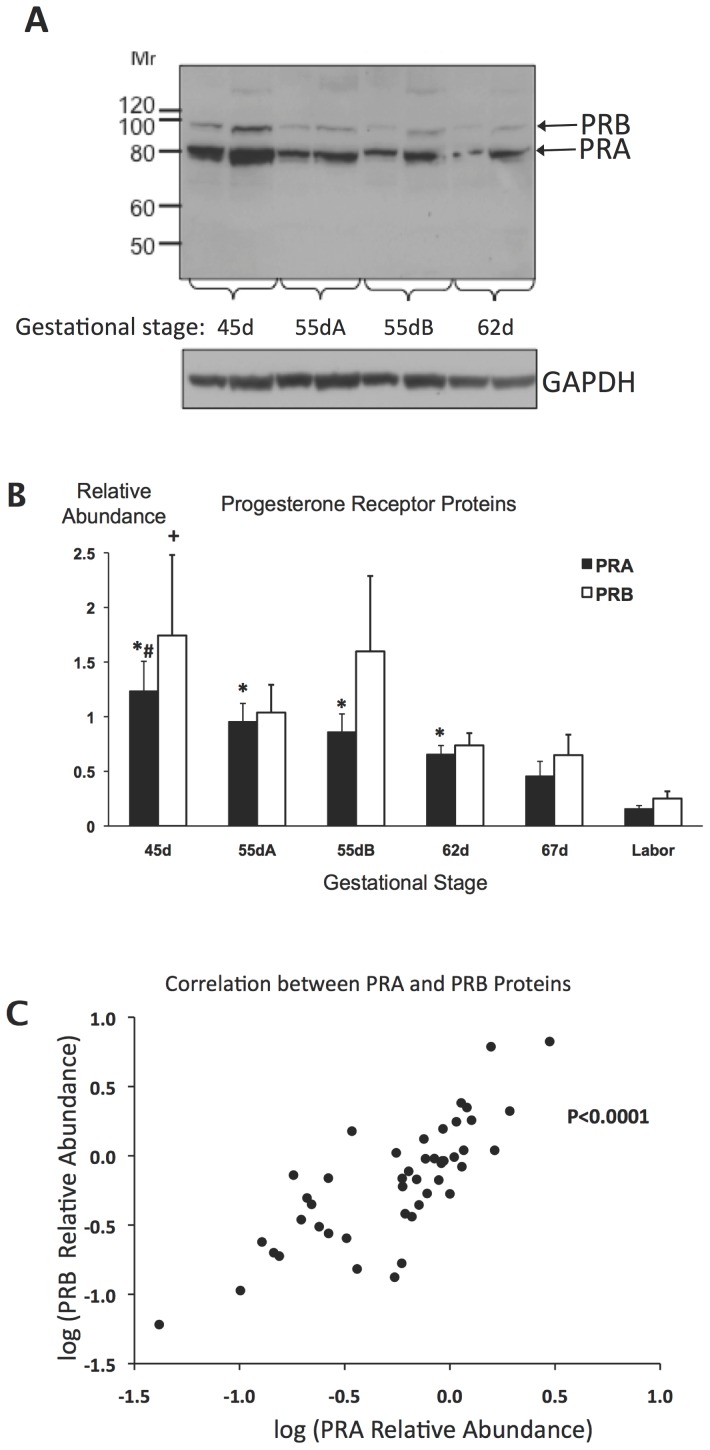
Progesterone receptor protein expression in guinea pig uteri during late gestation. **A**, immunoblot picture showing progesterone receptor A and B (PRA and PRB) and GAPDH loading control bands in one representative uterine sample of the gestational stage groups 45d, 55dA, 55dB and 62d. Duplicates of 50 µg (left) and 100 µg (right) protein content were run from each uterine extract. **B**, PRA and PRB protein relative abundance in the gestational stage groups from 45d pregnancy to Labor. *, PRA significantly different from 67d and Labor; #, PRA significantly different from 67d; **+**, PRB significantly different from Labor; p<0.05, ANOVA corrected for multiple comparisons (Bonferroni); n = 6–8 per group. **C**, Correlation between PRA and PRB relative abundance in individual uteri (p<0.0001, Spearman).

**Figure 2:**
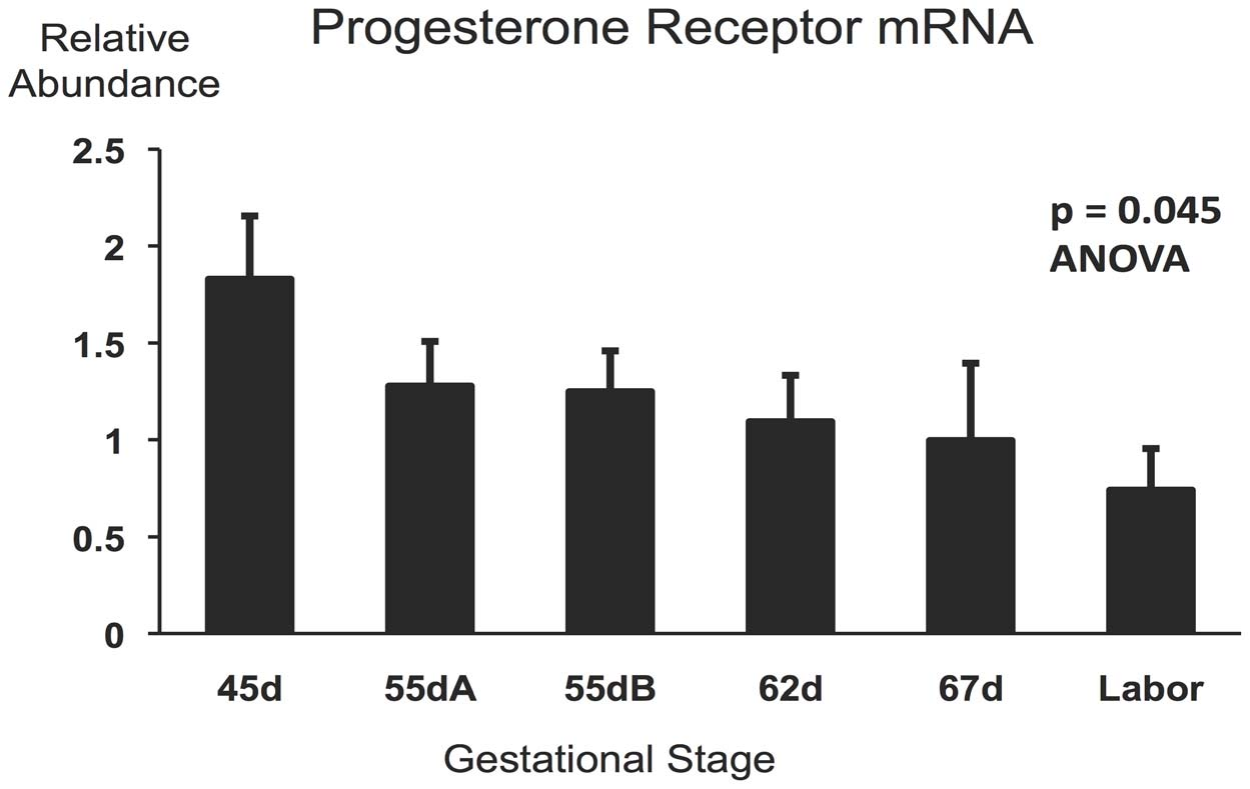
Progesterone receptor mRNA expression in guinea pig uteri during late gestation. Gestational stage groups (n = 6–8 per group) are described in the Methods. Overall significance is shown (ANOVA F = 2.52).

After electrophoresis proteins were electrotransferred to Hybond-P membrane (Amersham, GE Healthcare Ausrtalia, Rydalmere, NSW) in NuPAGE transfer buffer (containing 20% methanol) at 160 mA for 90 minutes. Uniform transfer was verified by Poinceau S staining (0.1% w/v in 5% v/v acetic acid). The membranes were blocked with 5% w/v skim milk in TBS (20 mM Tris HCl, pH 7.4; 0.9% w/v NaCl) at room temperature for 90 min. Primary antibodies were dissolved in blocking solution and incubated with the blots overnight at 4C. The following primary antibodies were used: Anti-progesterone receptor mouse monoclonal (clone PR-AT 4.14) from Thermo Scientific (Cat. No. MA1-410; Pierce Biotechnology) at 4 µg/ml; HC-20 (sc-543) anti-ESR1 rabbit polyclonal from Santa Cruz, 0.4 µg/ml; anti-GAPDH mouse monoclonal 6C5 (sc-32233) from Santa Cruz, at 1∶10000 dilution. The secondary antibodies were: anti-mouse IgG-HRP conjugate (#7076 Cell Signalling, Danvers, MA, USA) and anti-rabbit IgG-HRP conjugate (#7074, Cell Signalling, both at 1∶2000 dilution). Blots were incubated first with the steroid receptor antibodies, washed and incubated with the appropriate secondary antibodies at room temperature for 1 h. Bands were developed by enhanced chemiluminescence (Amersham ECL) following the supplier's instructions. Membranes were then dried, re-wetted and re-probed with the GAPDH primary antibody followed by the appropriate secondary antibody (anti-mouse IgG-HRP) and chemiluminescence detection. Chemiluminescent light was detected either by X-ray film (Figures 1A, S1 and Gels 1–13 in Figure S3A–C) or using a Fujifilm LAS 3000 Digital Imager (Figures 2A, S2, Gels 14–19 in Figure S3C–D, all gels in Figure S4A–C, S5, S6, S7). X-ray images were digitised using an UMAX PowerLook 1000 Scanner. Densitometric analysis and quantification were performed using the Multi Gauge Image analysis software (Fujifilm).

### RNA extraction, cDNA synthesis and real-time quantitative RT-PCR

Pulverised frozen uterine tissue was homogenised in Trizol (3 ml/0.2 g tissue) at 4C and extracts were prepared as instructed by the manufacturer (Invitrogen). Nucleic acid concentration was determined by UV absorption (NanoDrop, Thermo Scientific), and aliquots containing 60 µg were DNAse-treated and purified using the RNeasy Mini Kit (Qiagen Australia, Chadstone Centre, VIC). The integrity of the purified RNA was confirmed by agarose gel electrophoresis. Complementary DNA (cDNA) was synthetised using the Superscript III First-Strand System with random hexamer priming (Invitrogen). No-reverse transcriptase controls were generated with all RNA preparations.

PCR amplifications were performed using an Applied Biosystems 7500 Real Time PCR System. Each 25 µl reaction mixture contained PCR Master Mix with SYBR green, supplied by Applied Biosystems, cDNA template generated from 20 ng RNA and primers. Each sample was assayed in duplicate. Primer sequences were designed with Primer Express v3.0 (Applied Biosystems) and are listed in Table 1. Optimised primer concentrations were 1200 nM for PR, 1400 nM for *ESR1*, 500 nM for *ESR2* and 800 nM for *GAPDH* mRNAs. The reactions were performed in 98-well plates with thermal cycling conditions set by the manufacturer of the instrument (Applied Biosystems), followed by melt-curve analysis to ascertain the homogeneity of the amplification products. No-template and no-reverse transcriptase controls were included in each plate and with each cDNA sample, respectively. One cDNA sample was included in all plates to serve as calibrator to correct for plate-to-plate variation of amplifications.

**Table 1:** qRT-PCR primers.

Primer	Primer Sequence (5′ – 3′)	Amplicon length (bp)	Source
ESR1	Fwd.: TGATGGGCTTACTGACCAACCT	61	GenBank AY172106
	Rev.: TTCGCCCAGTTGATCATGTG		
ESR2	Fwd.: TCCCAGCAGCAGTCCATTC	81	GenBank AY172105
	Rev.: GGCTCTGGCTGGCTTCTG		
GAPDH	Fwd.: ACCTGCCGCCTGGAGAA	68	GenBank EU862201
	Rev.: CCCTCTGATGCCTGCTTCAC		
PR	Fwd.: GCAGGTCTACCCGCCCTATC	59	GenBank KJ563274
	Rev.: TTCGCCCAGTTGATCATGTG		

### Data analysis

#### Immunoblotting

Band intensity values were generated in arbitrary units by the image analysis software. Each band intensity value representing a target protein was divided by the corresponding calibrator value on the same blot to compensate for blot-to-blot variation. GAPDH was used as loading control; therefore receptor protein values were divided by the GAPDH value from the same sample to obtain the Relative Abundance values. Relative Abundance values were transformed to approach normal distribution if necessary. GAPDH values were compared between the gestational stage groups and showed no significant variation by one-way ANOVA, and there was no difference in GAPDH protein abundance between the gravid and non-gravid horns of the same uteri (Wilcoxon's matched-pairs rank-test) demonstrating consistent expression of the GAPDH loading control. Receptor protein Relative Abundance was compared between gestational stage groups using one-way ANOVA adjusted for multiple comparisons (Bonferroni). Receptor Relative Abundance in the gravid and non-gravid uterine horns was compared using the paired t-test, and drug *vs.* vehicle treatments were compared using the t-test or Mann-Whitney U-test, as appropriate. Correlation between PRA and PRB protein levels was assessed by the Spearman test.

#### qRT-PCR

The qRT-PCR systems were established, optimised and evaluated using the ΔΔCt method as described [17]. *GAPDH* mRNA was used as the reference. Messenger RNA Relative Abundance values were transformed to approach normal distribution and compared among the gestational groups by ANOVA with Bonferroni adjustment for multiple comparisons. *GAPDH* mRNA abundance, determined relative to the calibrator sample, was not different among the gestational groups by one-way ANOVA, indicating steady reference gene expression. *GAPDH* mRNA in the non-pregnant horns, however, was significantly less abundant than in the pregnant horns of the same uteri (Wilcoxon's matched-pairs rank-test); therefore, steroid receptor mRNA relative abundance values in the non-pregnant horns were corrected for the difference in the reference mRNA levels. Steroid receptor mRNA levels were compared between the pregnant and non-pregnant horns by Wilcoxon's matched-pairs rank-test. In all statistical analyses p<0.05 was considered significant.

## Results

### Progesterone receptor proteins and mRNA

PRA and PRB protein levels in guinea pig uterine tissues were determined by immunoblotting using a monoclonal antibody raised against a peptide (amino acids 533–546) of the human PR. Figure S1A shows a representative immunoblot image with two distinct bands corresponding to PRA and PRB at relative mobilities corresponding to 80,000 and 104,000 Da, respectively (Lane 3). The positive control, T47D cell extract, exhibited PRA and PRB protein bands with slightly lower relative mobilitites (85,000 and 109,000, respectively, Lane 2). All PR bands disappeared in the presence of excess immunising peptide during the immunoreaction demonstrating the specificity of the detection.

Figure 1A and the complete set of 19 blot images in Figures S3A–D show uterine PRA and PRB protein abundance in all guinea pigs in the gestational stage groups. The densitometric quantification of these immunoblots is presented in Table S1. One-way ANOVA indicated a significant decrease of both PRA (p<0.0001) and PRB (p<0.02) levels in the uterine tissues with gestation advancing from 45d to labor. The PRA and PRB protein levels were highly correlated in individuals throughout this period (p<0.0001, Figure 1C). The ratios of PRA to PRB protein abundance were not different between the gestational stage groups (not shown).

To measure *PR* mRNA levels in the guinea pig uterus, it was necessary to determine the nucleotide sequence, because this information was not available in the public databases. For this purpose, the human, mouse, rat and rabbit *PR* cDNA sequences were aligned and a highly conserved region was selected to design the following PCR primers using Primer3 [18]: 5′-TCCCGCAGCTCGGCTAC-3′, forward and 5′-AGGACCATGCCAGCCTGA-3′, reverse. Amplification of cDNA from guinea pig uterus by end-point PCR using these primers generated several products including one close to the expected size of 350 bp. This product was separated by agarose gel electrophoresis, purified and sequenced repeatedly on both strands. The following 342 bp consensus sequence was obtained:

5′CCCGCAGCTCGGCTACCAGGCCGCGGTGCTCAAGGACGGCCTGCCGCAGGTCTACCCGCCCTATCTCAACTACCTGAGACCGGATTCAGAAGCCAGCCAGAGCCCACAGTACAGCTTCGAGTCACTGCCTCAGAAGATTTGTCTTATCTGTGGTGATGAAGCATCAGGCTGTCATTATGGTGTCCTTACCTGTGGGAGCTGTAAGGTCTTCTTTAAAAGGGCGATGGAAGGGCAGCATAACTATCTATGTGCTGGAAGAAATGACTGCATTGTTGATAAAATCCGTAGAAAAAATTGCCCAGCATGTCGCCTTAGAAAATGCTGTCAGGCTGGCATGGTCCT-3′. This experimentally defined sequence was present within the computationally predicted Cavia Porcellus progesterone receptor mRNA sequence available in the NCBI database (accession number: XM_005004991.1). The NCBI Conserved Domain Search tool (http://www.ncbi.nlm.nih.gov/Structure/cdd/wrpsb.cgi. Accessed 2014 July 24) generated two major hits on this nucleotide sequence. One was the shared DNA-binding domain of the nuclear glucocorticoid and progesterone receptors (E-value: 3.41e-61) and the other was an adjacent conserved progesterone receptor domain (E-value: 1.06e-26). Based on this information we concluded that we have obtained a guinea pig *PR* partial cDNA sequence corresponding to a region common to *PRA* and *PRB*. The sequence has been deposited in GeneBank (accession number: KJ563274). Using the specific progesterone receptor domain sequence we have designed qRT-PCR primers for *PR* mRNA determination. The sequences of the primers are in Table 1. The measurement of *PR* mRNA abundance in total RNA from uterine tissues of the different gestational stage groups showed a significant overall decrease of *PR* mRNA expression (p = 0.045, ANOVA, n = 46) as gestation progressed from 45d to labor (Figure 2). There was no statistically significant correlation between *PR* mRNA abundance and either PRA of PRB protein levels in individuals (after adjusting for multiple comparisons) suggesting that PR protein levels were strongly influenced by receptor protein turnover.

### Estrogen receptor protein and mRNA

ESR1 protein was detected by immunoblotting using the rabbit polyclonal antibody ERα (HC-20). The antibody produced several immunoreactive bands in the positive control (T47D cell extract) including one with the expected relative mobility of ESR1 (corresponding to 66,000 Da; Figure S2, Lane 4). In the guinea pig uterine tissue extract this band was undetectable (Figure S2, Lane 3). Ammonium sulphate fractionation of the uterine extracts at 33% saturation, which is known to precipitate steroid receptor proteins, resulted in a protein fraction that contained detectable amounts of ESR1 (Figure S2, Lane 2). Excess immunising peptide blocked the ESR1 band (Figure S2, Lanes 6 and 8), confirming that the antibody reacted with the guinea pig ESR1 protein.

**Figure 3:**
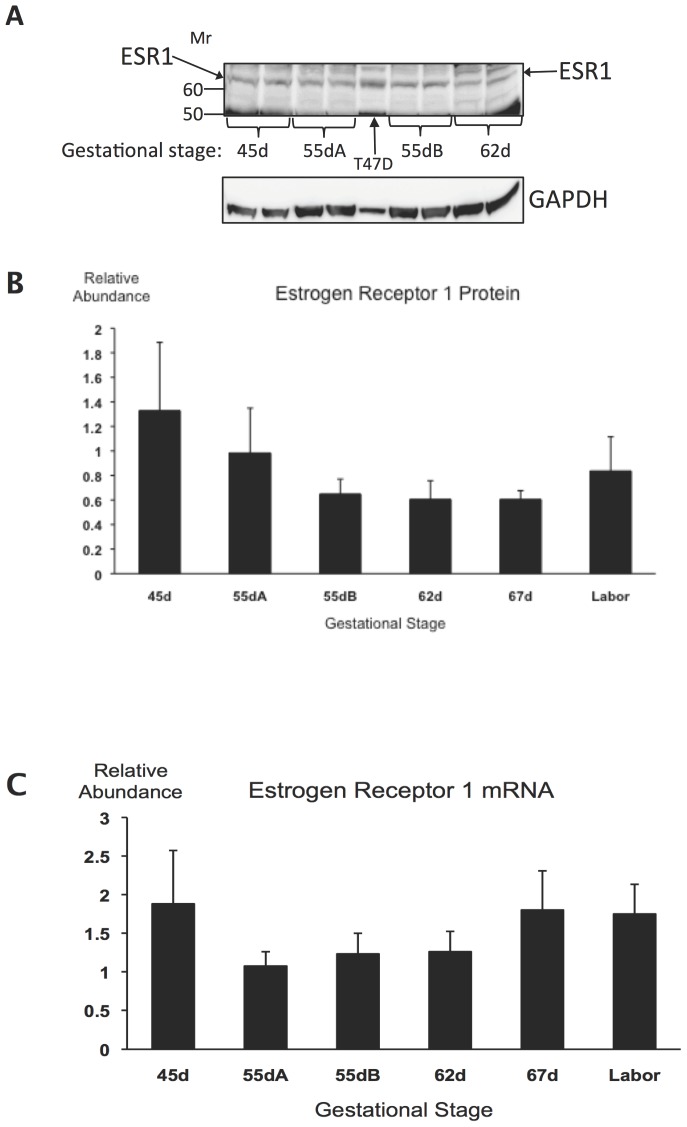
Estrogen receptor alpha (ESR1) expression in guinea pig uteri during late pregnancy. **A**, Representative immunoblot pictures showing ESR1 protein and GAPDH loading control in one representative uterine sample of the gestational stage groups 45d, 55dA, 55dB and 62d. The T47D cell extract is positive control. Duplicates containing 60 µg protein were run from each uterine extract. **B**, ESR1 protein relative abundance determined by densitometry. Variance is not significantly different among the gestational stage groups. **C**, *ESR1* mRNA relative abundance in guinea pig uteri at late gestation. Variance is not significantly different among the gestational stage groups (n = 6–8 per group).

Estrogen receptor 1 protein was detected in all uterine samples, as shown in the representative blot image in Figure 2A and in the full set of 14 images in Figures S4A–C. Densitometric analysis results are presented in Table S2. There was no significant change with advancing gestation (ANOVA, Figure 3B). Estrogen receptor 1 mRNA relative abundance was also measured in the same tissue samples using qRT-PCR and was found not to change between 45d and Labor (Figure 3C). There was no significant correlation between ESR1 protein and mRNA levels in individuals. We have obtained qRT-PCR primers for detecting guinea pig *ESR2* mRNA (Table 1), but the mRNA of this estrogen receptor isoform was undetectable in all uterine tissue samples.

### Differences between the gravid and non-gravid uterine horns

**Figure 4:**
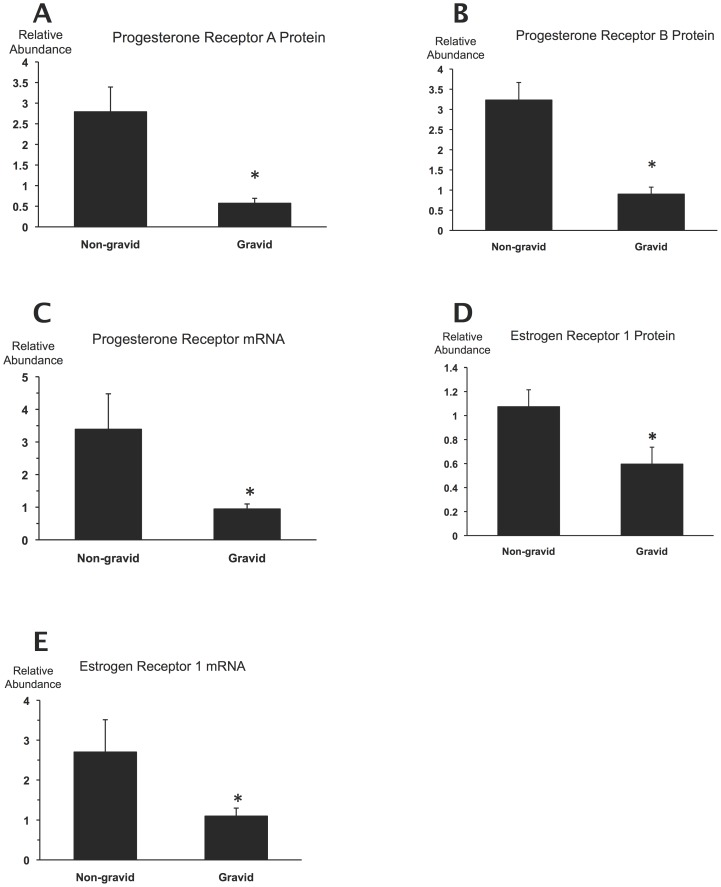
Receptor expression in the gravid and non-gravid uterine horns. **A**–**E**, Progesterone receptor A and B protein, mRNA and Estrogen receptor (ESR1) protein and mRNA relative abundance in the gravid and non-gravid horns of the same uteri (n = 9). *, p<0.05 relative to the non-gravid horn determined by the paired t-test and Wilcoxon's matched-pairs signed-rank test with normally and non-normally distributed data, respectively.

Occasionally, one horn of the bicornuate uterus contains all fetuses in a pregnant guinea pig. This provides opportunity to examine the gravid and non-gravid horns separately and assess the local effects of the gestational sac on the gravid uterine horn in contrast to the non-gravid horn, which is not in contact with fetal tissue. In this study we have encountered 9 pregnancies with only one gravid horn of which 3 were from Group 55dB, 3 from Group 62d, 2 from Group 67d and 1 from Labor. As shown in Figure 4, PRA, PRB and ESR1 protein and *PR* and *ESR1* mRNA levels were significantly higher in the non-gravid than in the gravid horns. (Immunoblot images and densitometric analysis results generated by the non-gravid horns are included in Figures S3, S4 and in Tables S1, S2, respectively). The number of unilateral pregnancies was too low in our guinea pig cohort to determine changes that may have occurred during gestation; however it is clear that the presence of the gestational sac was associated with lower receptor expression in the surrounding uterine tissue.

### The effects of prostaglandin administration on progesterone and estrogen receptor expression

We have explored the possibility that prostaglandin administration to guinea pigs changes PR isoform expression in the uterus using Sulprostone, a non-metabolising PGE_2_ analogue, which is a potent abortifacient stimulating fetal expulsion at any time during pregnancy [11]. Our Sulprostone treatment protocol caused incomplete abortion in two of the eight animals; therefore it modelled late gestation exposure to prostaglandin without interference by post-partum changes. The results presented in Table 2 show that Sulprostone caused a significant decrease of PRA protein and *PR* mRNA levels in the uterine tissues, while the median PRB protein abundance was not significantly different. *ESR1* mRNA abundance was also decreased significantly by Sulprostone; however, there was no concomitant decrease in ESR1 protein levels. PRA, PRB and ESR1 immunoblot images of the complete Sulprostone study are presented in Figures S5 and S6, respectively. Densitometric results are presented in Table S3.

**Table 2:** The effects of Sulprostone on PRA, PRB and ESR1 protein and PR and ESR1 mRNA Relative Abundance in guinea pig uteri.

Vehicle						Sulprostone					Significance
Receptor	N	Mean	SD	Median	Range	N	Mean	SD	Median	Range	**P**
PRA protein	8	1.43	0.45	1.22	0.95–2.07	8	0.901	0.33	0.99	0.32–1.36	**0.016^b^**
PRB protein	8	0.88	0.39	0.78	0.51–1.68	8	0.66	0.26	0.60	0.28–1.06	0.24^a^
PR mRNA	8	1.32	0.66	1.15	0.67–2.31	8	0.66	0.37	0.67	0.13–0.68	**0.023^b^**
ESR1 protein	8	20.77	28.2	9.65	3.05–86.2	8	17.9	30.0	2.43	0.47–87.8	0.21^a^
ESR1 mRNA	8	1.08	0.47	1.01	0.61–1.95	8	0.43	0.17	0.36	0.24–0.68	**0.003** a

Details of the treatments are described in the text.

a , Mann-Whitney test; ^b^, two-sample t-test.

Significant differences are in bold. N: number of animals per group.

### The effect of prostaglandin synthesis inhibitor on progesterone and estrogen receptor expression

We have examined the involvement of endogenous prostaglandins in the control of uterine progesterone and estrogen receptor expression by treating pregnant guinea pigs with the prostaglandin synthase inhibitor Piroxicam using a treatment regimen shown previously to prolong gestation [13]. As shown in Table 3, PRA protein abundance was significantly higher in uterine tissues after Piroxicam treatment compared to vehicle. PRB and ESR1 protein levels and *PR* mRNA and *ESR1* mRNA abundance did not change significantly in response to Piroxicam. PRA, PRB and ESR1 immunoblots of the complete Piroxicam study are shown in Figure S7, and the corresponding densitometric analysis results are in Table S4.

**Table 3:** The effects of Piroxicam on PRA, PRB and ESR1 protein and PR and ESR1 mRNA Relative Abundance in guinea pig uteri.

Vehicle						Piroxicam					Significance
Receptor	N	Mean	SD	Median	Range	N	Mean	SD	Median	Range	**P**
PRA protein	7	1.11	0.26	1.09	0.82–1.56	8	1.66	0.55	1.58	1.16–2.77	**0.015** a
PRB protein	7	1.58	0.55	1.38	1.0–2.46	8	1.96	0.83	1.79	0.91–3.32	0.31^b^
PR mRNA	7	0.81	0.30	0.86	0.21–1.16	8	0.77	0.66	0.66	0.22–2.18	0.64^a^
ESR1 protein	7	0.28	0.17	0.29	0.13–0.61	8	0.38	0.33	0.23	0.13–0.99	0.86^a^
ESR1 mRNA	7	1.60	0.61	1.01	0.55–2.29	8	1.20	0.24	1.26	0.75–1.50	0.13^b^

Details of the treatments are described in the text.

a , Mann-Whitney test; ^b^, two-sample t-test.

Significant difference is in bold. N: number of animals per group.

## Discussion

The declining progesterone responsiveness of the myometrium is a key component of the birth process in women, who progress to labor and deliver in the presence of high circulating progesterone concentrations. The causes of the diminishing hormone responsiveness are unclear, but one possibility is that the molecular apparatus mediating progesterone action in the myometrial cells changes at term decreasing the efficacy of the hormone to maintain uterine quiescence and cervical closure [1]. The nuclear receptors PRA and PRB mediate the effects of progesterone on gene expression, and progesterone receptor concentrations are major determinants of the progesterone responsiveness of the target tissues. Observational studies by Mesiano et al., have shown that PRA and PRB mRNA and protein expression is altered in the human uterus at labor resulting in an increase of the ratio of PRA to PRB levels [6], [19]. PRA is a ligand-dependent transdominant repressor of PRB [20] and PRA stimulates labor-promoting inflammatory gene expression in myometrial cells [21] suggesting that a shift towards increased PRA:PRB ratio may cause a drop in the ability of progesterone to maintain uterine quiescence. Furthermore, there is strong positive correlation between the PRA:PRB ratio and *ESR1* mRNA abundance and between *ESR1* mRNA abundance and prostaglandin synthase-2 (*PTGS2*) and oxytocin receptor (*OTR*) mRNA levels in term human uterine tissue [6]. Based on these observations it was proposed that labor in women was associated with functional progesterone withdrawal combined with estrogen activation at the receptor level leading to estrogen dominance and the induction of contraction associated proteins such as PTGS2 and OTR.

Prostaglandins, especially PGE_2_ and PGF_2α_, induce abortion and birth at any time during gestation in mammals [11]. The principal mode of action of PGF_2α_ in mice is to induce luteolysis with the consequent decline of maternal plasma progesterone levels followed by the increase of OTR and other contraction-associated protein expression in the myometrium [22]. In previous work we have explored the possibility that, by analogy, prostaglandins may also be involved in the mechanism of functional progesterone withdrawal. We found that the treatment of immortalised human pregnant myometrial cells (the PHM1-31 cell line) with PGE_2_ or PGF_2α_ increased the *PRA:PRB* mRNA expression ratio mimicking the change occurring in human uterine tissue at labor [9]. Prostaglandins, therefore, may have the potential to cause functional progesterone withdrawal as part of their action promoting labor.

In the present investigation we used the guinea pig to study the involvement of steroid receptor regulation in functional progesterone withdrawal and the role of prostaglandins in the process in intact pregnant animals in vivo. Progesterone withdrawal at term is functional in guinea pigs and is concomitant with increased prostaglandin synthesis by the amnion membrane, as in women [4], [23] [13]. We have collected uteri between 45d of pregnancy and labor, which corresponds to the last third of gestation (delivery occurs between 68–71 days of pregnancy in our colony) and found a significant decrease of both PRA and PRB protein abundance with approaching term. The levels of the two PR isoforms correlated strongly in individual uteri suggesting that the decrease took place in a coordinated fashion. Our *PR* mRNA qRT-PCR assay was not isoform-selective and measured the sum of *PRA* and *PRB* mRNAs, which also decreased significantly with advancing gestation. The *PR* mRNA level, however, showed no significant correlation with either PRA or PRB protein abundance in individual uteri indicating that PR receptor expression was controlled predominantly by receptor protein turnover rather than PR gene activity. Further work is needed to elucidate the exact molecular mechanisms regulating PR receptor levels in the uterus.

Decreased PRA *and* PRB expression, also observed in experimentally induced growth restriction in guinea pig pregnancies prone to preterm birth [24], is a plausible mechanism of functional progesterone withdrawal. It is in agreement with earlier work showing a decrease of high affinity progesterone binding in nuclei from term guinea pig myometrium [16]. Recent results also support declining PR expression as a mechanism of functional progesterone withdrawal in guinea pig cervix [25], [26]. Thus functional progesterone withdrawal in guinea pigs appears receptor-mediated like in women; however, the exact mechanism is different, since it manifests as a decrease in the abundance of both PR isoforms and not as an increase of the PRA:PRB ratio. The two mechanisms can produce the similar end result representing analogous traits that may have evolved in the two species in a convergent fashion, as suggested by the high-throughput transcriptome analysis of guinea pig cervix [25].

Uterine ESR1 expression remained steady during the last third of guinea pig pregnancy, which in view of the decreasing PR levels suggests that estrogen dominance develops in the uterus even in the absence of increasing maternal estrogen concentration at term [4]. This represents further analogy with human pregnancy where estrogen dominance develops in the myometrium without a labor-associated rise in maternal plasma estrogen concentration, but supported by the increase of ESR1 expression [6].

An important aim of this study was to explore whether labor-inducing prostaglandins can promote functional progesterone withdrawal. This possibility was demonstrated by cell culture experiments, but in vivo testing required an animal model exhibiting a receptor-mediated decrease of myometrial progesterone responsiveness at term. Our results show that Sulprostone, a PGE-analogue with high potency to induce parturition in guinea pigs [11], caused a significant decrease of PRA protein and *PR* mRNA abundance in the uterus at 45d of pregnancy imitating the changes that occur before normal birth. PRB protein level remained unchanged, however; which may have been due to the relatively low Sulprostone dose (0.25–0.34 mg/kg), which caused partial abortion in 2 animals and no delivery in the remaining 6 in the group. ESR1 protein levels were unaffected in the Sulprostone treated animals. The importance of PRA for maintaining the progesterone responsiveness of guinea pig myometrium is further highlighted by the effect of Piroxicam, a PG-synthesis inhibitor drug. A Piroxicam treatment regimen shown previously to prolong guinea pig gestation and block the prostaglandin synthase enzyme [13] increased PRA protein abundance in the uterus, but did not affect the levels of PRB and ESR1. Collectively, our experiments using a PGE analogue and a PG-synthesis inhibitor strongly suggest that labor-inducing prostaglandins promote functional progesterone withdrawal and estrogen dominance in the myometrium in vivo by altering PRA, but not ESR1, expression.

The robust difference of all measured progesterone and estrogen receptor expression parameters between the pregnant and non-pregnant uterine horns indicates that the fetus suppresses progesterone and estrogen receptor expression in its proximity. This local effect may be mediated by prostaglandins produced by the gestational tissues [12], and/or by distension caused by the growing fetus [27]. We did not encounter high enough number of unilateral pregnancies to determine gestational changes in receptor levels in the non-pregnant horns, but a recent study using experimentally growth restricted pregnant animals detected a decreasing trend of PR expression in both the gravid and the non-gravid uterine horns at late gestation [24]. Moreover, Rodriguez et al. reported decreasing PR expression at term in the upper segment of uterine horns where no fetuses are implanted [28], which may also indicate the existence systemic suppressive factors still to be identified.

It is to be noted that our PR and ER mRNA and protein measurements determined global levels in the uterus and did not differentiate between the various uterine cell types. This analysis has been performed previously by Rodriguez et al., who have shown by immunohistochemistry that PRs and ER1 are localised to the myometrial and subepithelial layers of the guinea pig uterus [26], [28]. Thus, progesterone and estrogen can influence gene expression directly in the myometrial cells, but the contribution of the subepithelial connective tissue to the steroid regulation of uterine function remains to be determined.

In conclusion, the present findings demonstrate that decreasing uterine expression of both PRA and PRB is a mechanism of functional progesterone withdrawal in the guinea pig at term parturition. Our observations also indicate that labor-stimulating prostaglandins down-regulate PRA expression, which may reduce uterine progesterone responsiveness and cause functional progesterone withdrawal in the guinea pig. The receptor-mediated control of progesterone responsiveness is an attribute analogous with humans, highlighting the usefulness of the guinea pig for studies to explore the hormonal regulation of birth in experimental settings not possible with women.

## Supporting Information

Figure S1 **Immunoblot detection of progesterone receptor (PRA and PRB) proteins in guinea pig uterus.**
(PDF)

Figure S2 **Immunoblot detection of estrogen receptor (ESR1) protein in guinea pig uterus.**
(PDF)

Figure S3 **Panels A–D; progesterone receptor and GAPDH loading control immunoblots for determining PRA and PRB protein levels in guinea pig uterus.**
(PDF)

Figure S4 **Panels A–C; estrogen receptor (ESR1) and GAPDH loading control immunoblots for determining ESR1 protein levels in guinea pig uterus.**
(PDF)

Figure S5 **Immunoblots for determining the effect of Sulprostone on PRA and PRB protein levels in guinea pig uterus.**
(PDF)

Figure S6 **Immunoblots for determining the effect of Sulprostone on ESR1 protein levels in guinea pig uterus.**
(PDF)

Figure S7 **Immunoblots for determining the effect of Piroxicam on PRA, PRB, and ESR1 protein levels in guinea pig uterus.**
(PDF)

Table S1 **Densitometric evaluation of progesterone receptor immunoblots presented in Figure S3.**
(PDF)

Table S2 **Densitometric evaluation of estrogen receptor (ESR1) immunoblots presented in Figure S4.**
(PDF)

Table S3 **Densitometric evaluation of progesterone receptor and ESR1 immunoblots presented in Figure S5 and Figure S6, respectively (Sulprostone study).**
(PDF)

Table S4 **Densitometric evaluation of progesterone receptor and ESR1 immunoblots presented in Figure S7 (Piroxicam study).**
(PDF)
