# Supplementary material for: Progesterone Receptor Expression Declines in the Guinea Pig Uterus during Functional Progesterone Withdrawal and in Response to Prostaglandins
Source: PLoS One. 2014 Aug 26;9(8):e105253. doi: 10.1371/journal.pone.0105253 (PMC4144885; doi:10.1371/journal.pone.0105253)
Supplement: Figure S1 — Immunoblot detection of progesterone receptor (PRA and PRB) proteins in guinea pig uterus. (PDF) [file pone.0105253.s001.pdf]

Figure S1
Immunoblot Detection of Progesterone Receptor (PRA and PRB) Protein in Guinea Pig Uterus

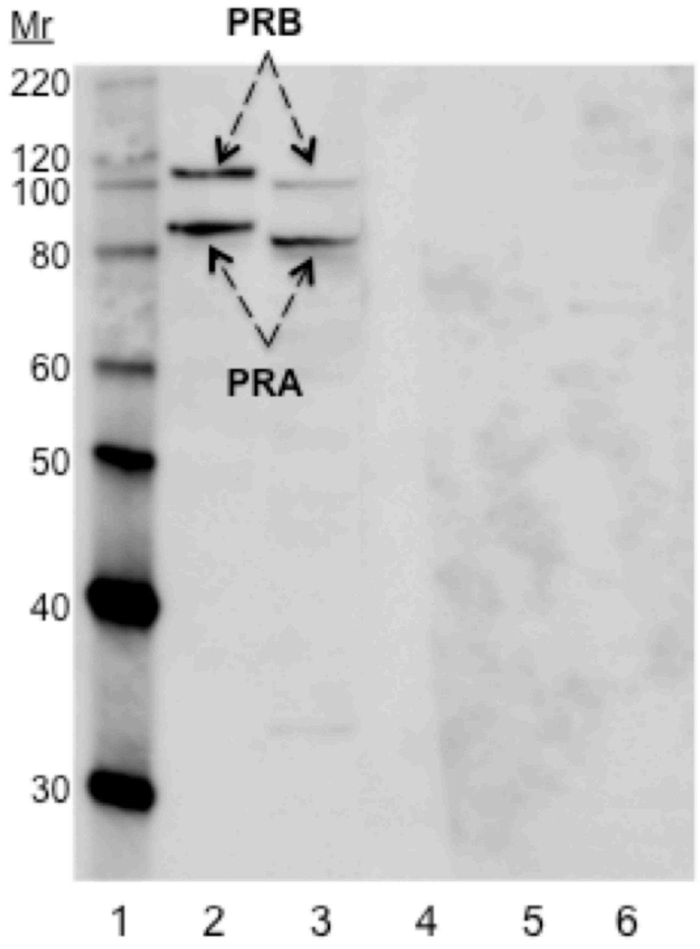

Lane Assignments:

1: Mr markers

2: T47D cell extract, positive control

3: Uterine tissue extract

4 : Empty lane

5: T47D cell extract with immunising peptide competition

6: Uterine tissue extract with immunising peptide competition
