# Supplementary material for: Progesterone Receptor Expression Declines in the Guinea Pig Uterus during Functional Progesterone Withdrawal and in Response to Prostaglandins
Source: PLoS One. 2014 Aug 26;9(8):e105253. doi: 10.1371/journal.pone.0105253 (PMC4144885; doi:10.1371/journal.pone.0105253)
Supplement: Figure S2 — Immunoblot detection of estrogen receptor (ESR1) protein in guinea pig uterus. (PDF) [file pone.0105253.s002.pdf]

Figure S2
Immunoblot Detection of Estrogen Receptor (ESR1) Protein in Guinea Pig Uterus

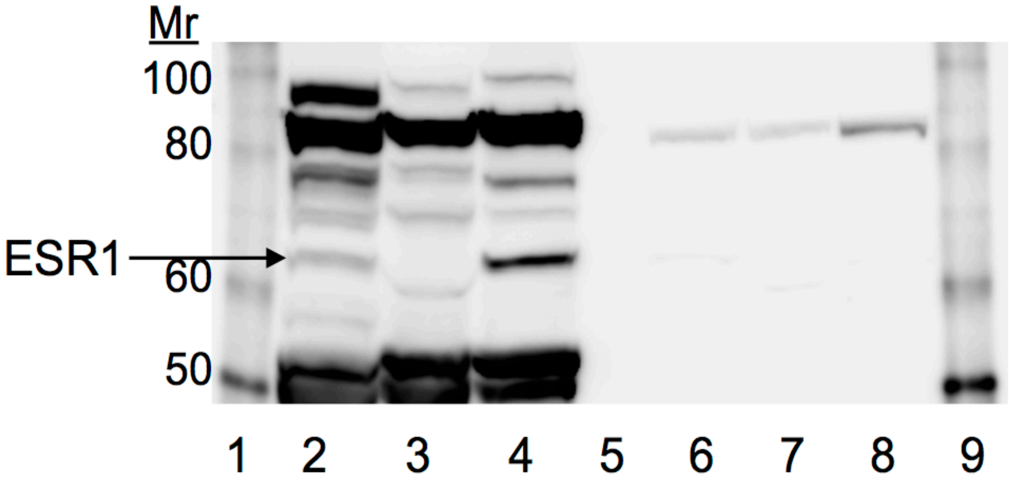

Lane Assignments:

1: Mr markers

2: Uterine tissue extract precipitated with ammonium sulphate

3: Uterine tissue extract

4 : T47D cell extract (positive control)

5: Empty lane

6: Uterine tissue extract precipitated with ammonium sulphate and immunising peptide competition

7: Uterine tissue extract with immunising peptide competition

8: T47D cell extract with immunising peptide competition

9: Mr markers
