# Supplementary material for: Progesterone Receptor Expression Declines in the Guinea Pig Uterus during Functional Progesterone Withdrawal and in Response to Prostaglandins
Source: PLoS One. 2014 Aug 26;9(8):e105253. doi: 10.1371/journal.pone.0105253 (PMC4144885; doi:10.1371/journal.pone.0105253)

Figure S3 A

Uterine PRA and PRB Protein Levels During Pregnancy

(Key to lane assignments is in Table S1)

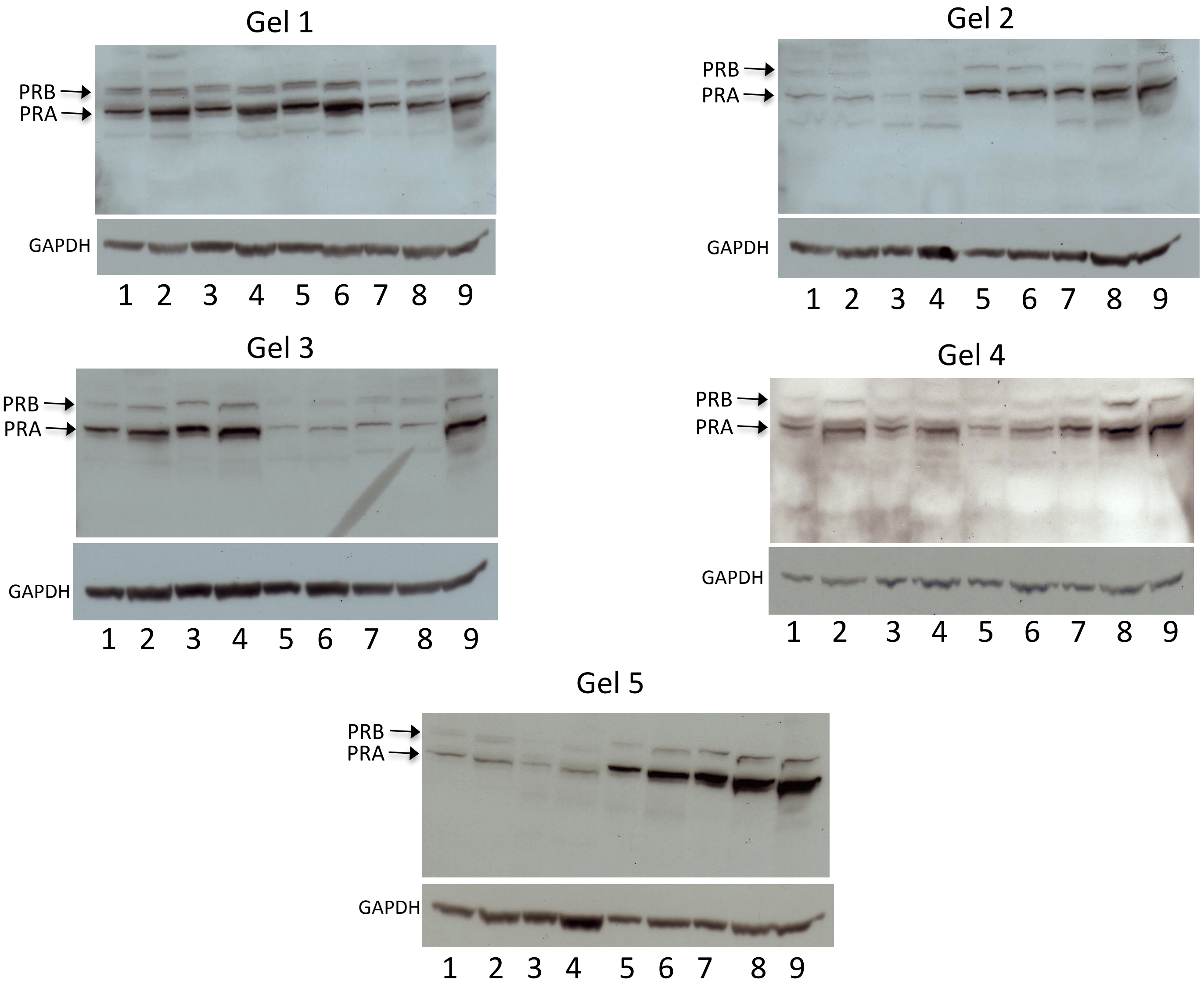

Figure S3 B
Uterine PRA and PRB Protein Levels During Pregnancy
(Key to lane assignments is in Table S1)

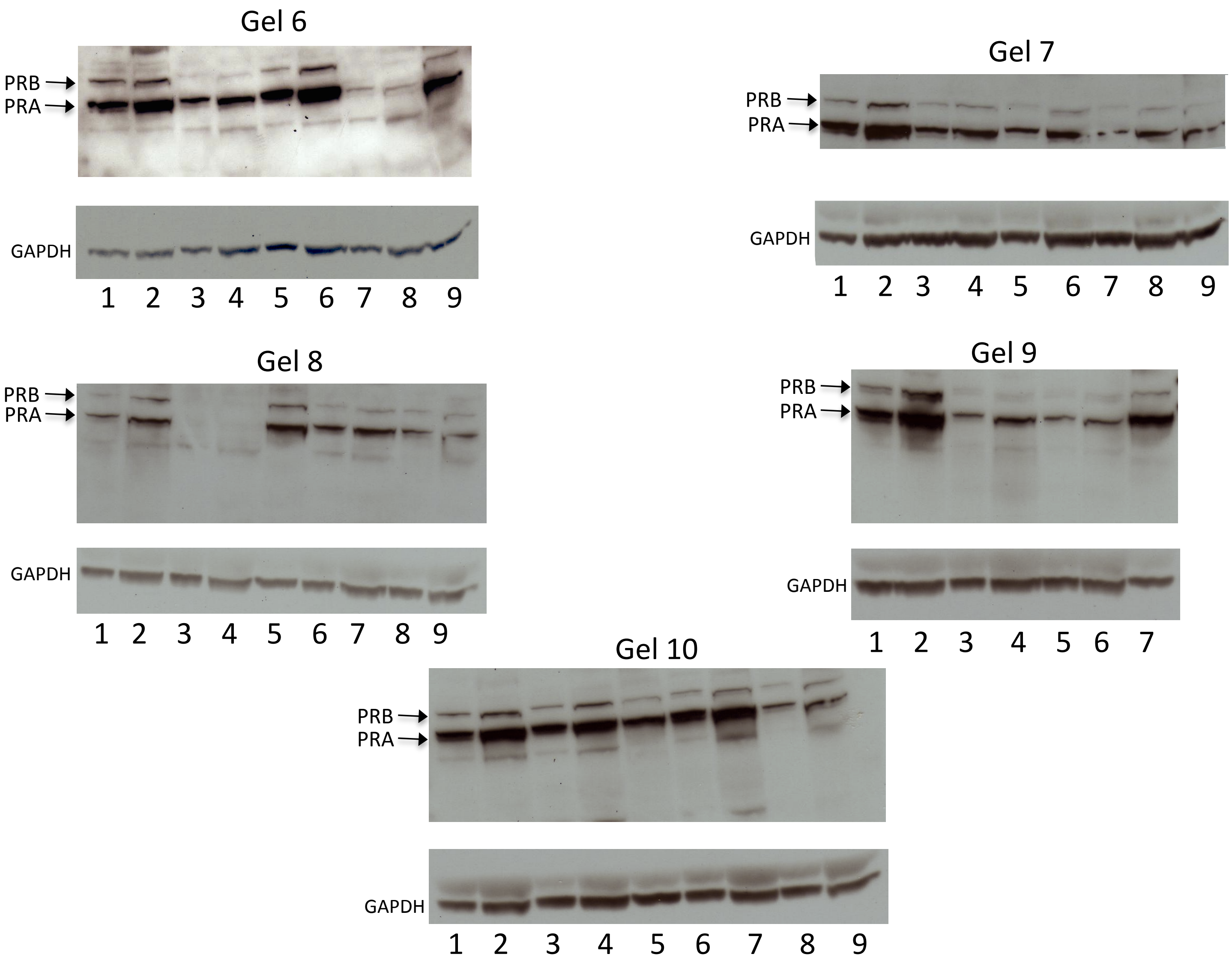

Figure S3 C
Uterine PRA and PRB Protein Levels During Pregnancy
(Key to lane assignments is in Table S1.)

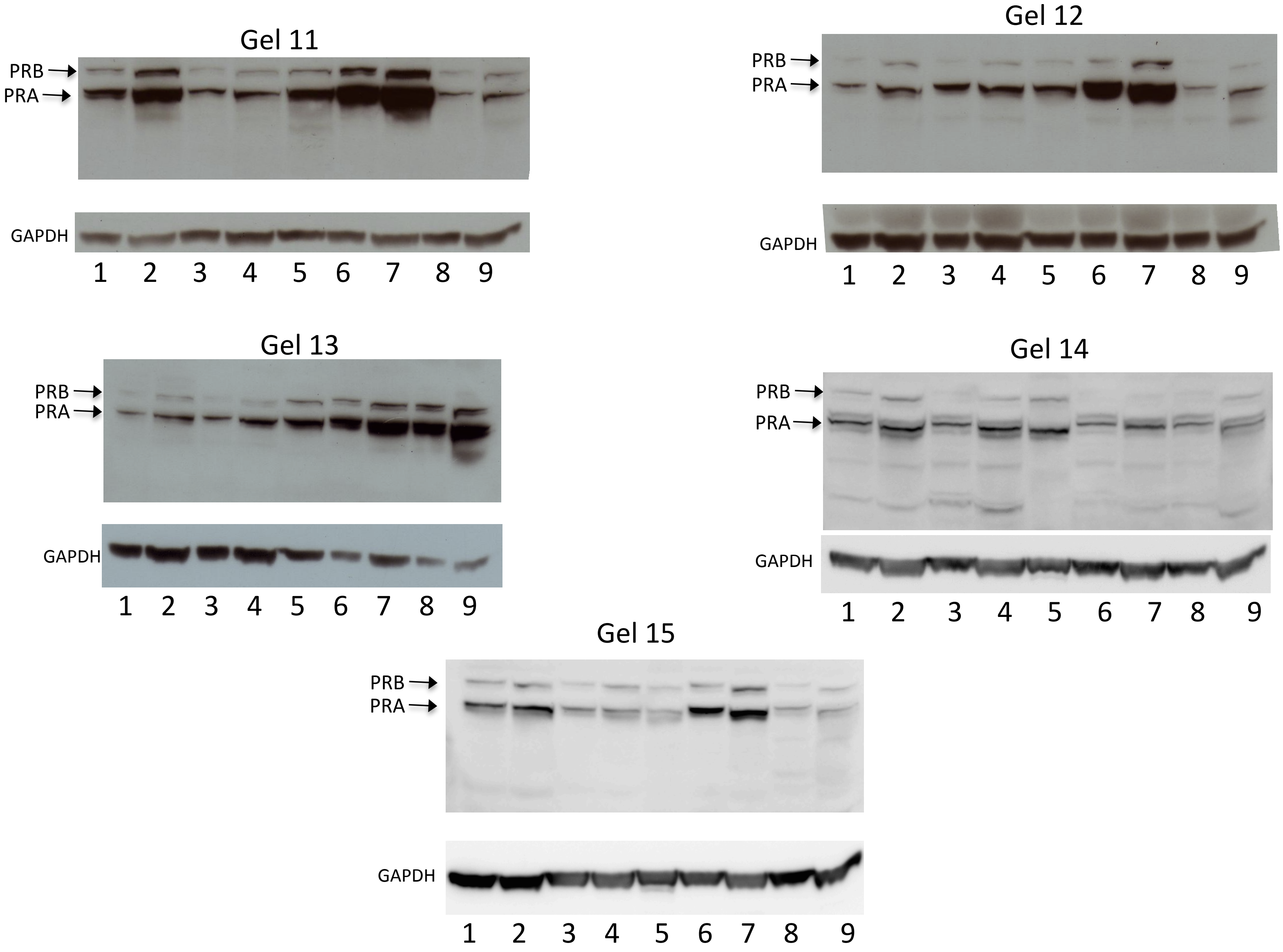

Figure S3 D
Uterine PRA and PRB Protein Levels During Pregnancy
(Key to lane assignments is in Table S1.)

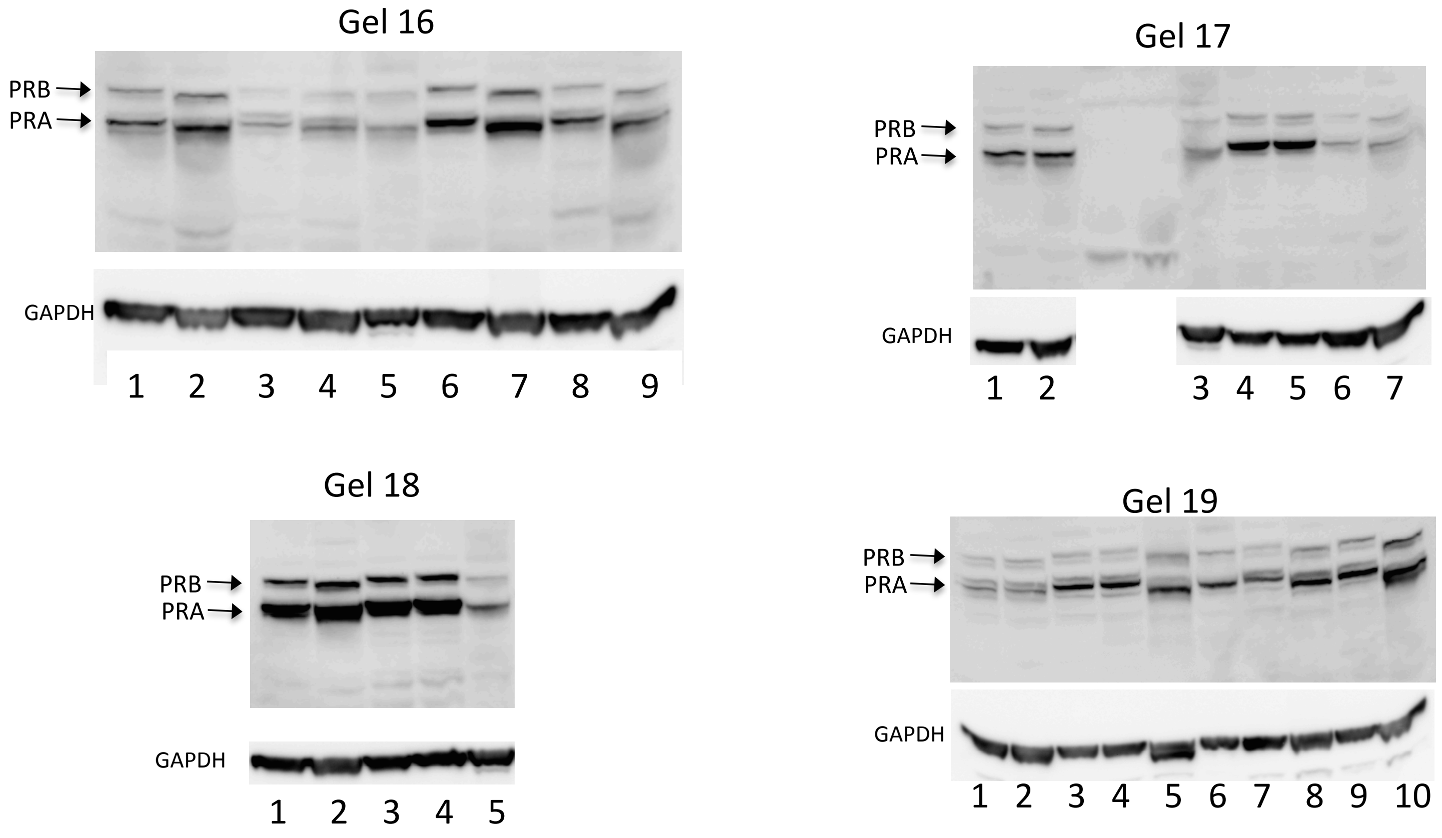

Supplement: Figure S3 — Panels A–D; progesterone receptor and GAPDH loading control immunoblots for determining PRA and PRB protein levels in guinea pig uterus. (PDF) [file pone.0105253.s003.pdf]
