# Supplementary material for: Progesterone Receptor Expression Declines in the Guinea Pig Uterus during Functional Progesterone Withdrawal and in Response to Prostaglandins
Source: PLoS One. 2014 Aug 26;9(8):e105253. doi: 10.1371/journal.pone.0105253 (PMC4144885; doi:10.1371/journal.pone.0105253)

Figure S4 A: Uterine ESR1 Protein Levels During Pregnancy

(Lane assignments are described in Table S2.)

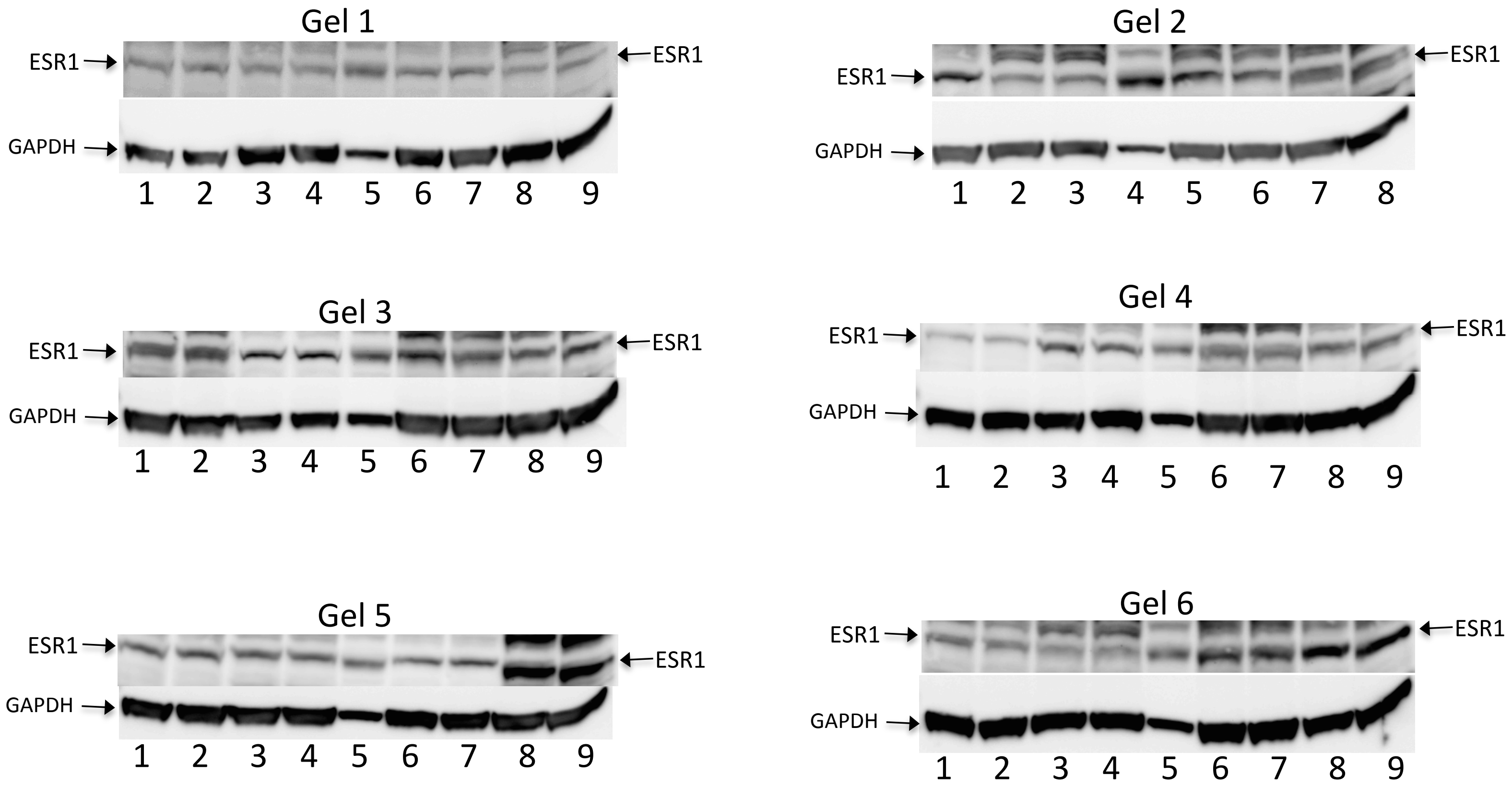

Figure S4 B: Uterine ESR1 Protein Levels During Pregnancy

(Lane assignments are described in Table S2 .)

Gel 7

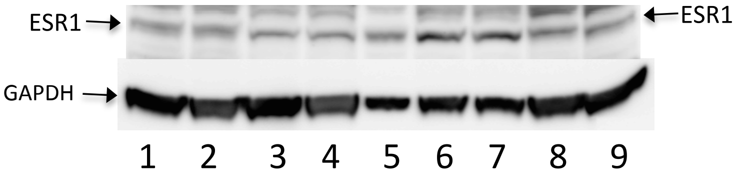

Gel 8

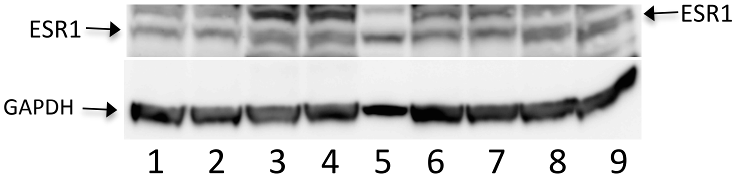

Gel 9

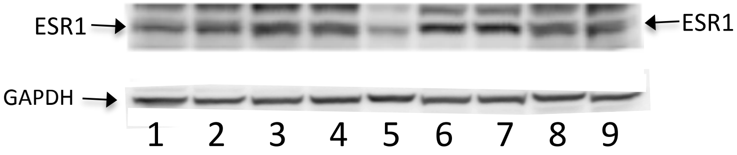

Gel 10

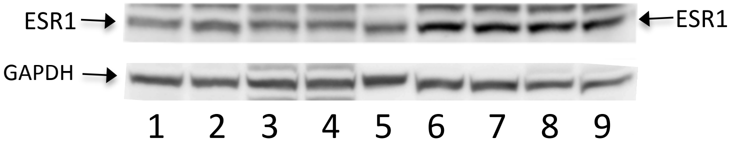

Gel 11

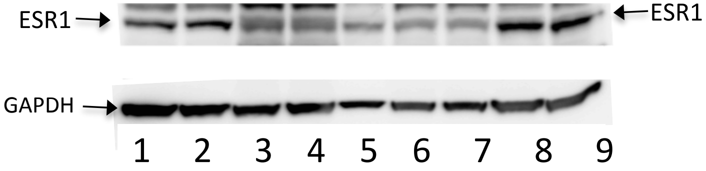

Gel 12

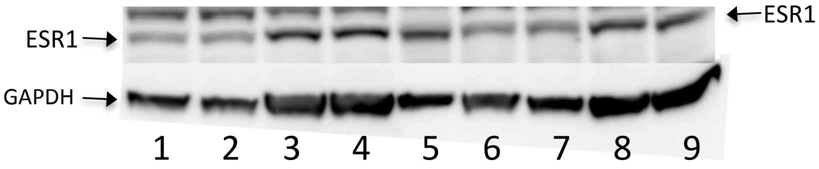

Figure S4 C: Uterine ESR1 Protein Levels During Pregnancy

(Lane assignments are described in Table S2.)

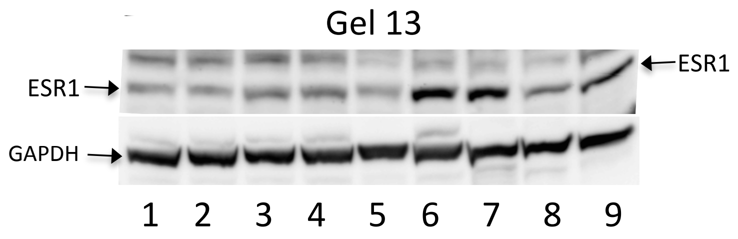

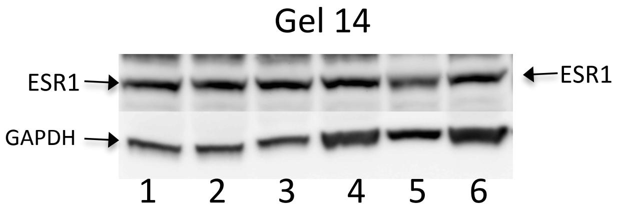

Supplement: Figure S4 — Panels A–C; estrogen receptor (ESR1) and GAPDH loading control immunoblots for determining ESR1 protein levels in guinea pig uterus. (PDF) [file pone.0105253.s004.pdf]
