# Supplementary material for: Progesterone Receptor Expression Declines in the Guinea Pig Uterus during Functional Progesterone Withdrawal and in Response to Prostaglandins
Source: PLoS One. 2014 Aug 26;9(8):e105253. doi: 10.1371/journal.pone.0105253 (PMC4144885; doi:10.1371/journal.pone.0105253)

Fig. S6: Effect of Sulprostone on Uterine ESR1 Protein Levels

(Lane assignments are described in Table S3.)

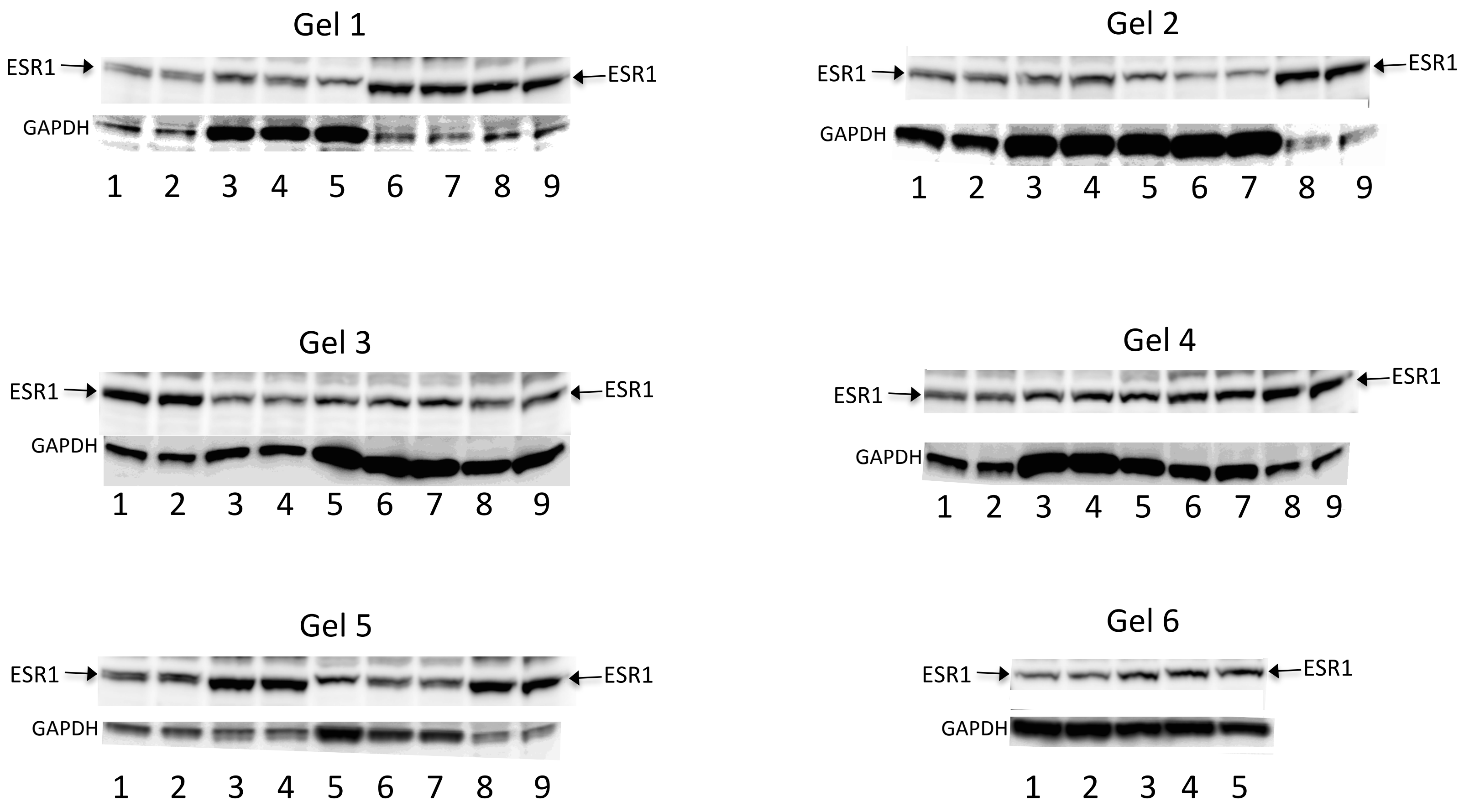

Supplement: Figure S6 — Immunoblots for determining the effect of Sulprostone on ESR1 protein levels in guinea pig uterus. (PDF) [file pone.0105253.s006.pdf]
