# Supplementary material for: Progesterone Receptor Expression Declines in the Guinea Pig Uterus during Functional Progesterone Withdrawal and in Response to Prostaglandins
Source: PLoS One. 2014 Aug 26;9(8):e105253. doi: 10.1371/journal.pone.0105253 (PMC4144885; doi:10.1371/journal.pone.0105253)
Supplement: Table S1 — Densitometric evaluation of progesterone receptor immunoblots presented in Figure S3. (PDF) [file pone.0105253.s008.pdf]

Table S1

Guinea Pig Uterine Progesterone Receptor Protein (PRA and PRB) Levels During Pregnancy

(Arbitrary Densitometric Units)

Gel 1

Lane	Sample ID and protein loading	PR-A	PR-B	PR-A/PR-B	GAPDH	PR-A/GAPDH	Rel to CAHm*	PR-B/GAPDH	Rel to CAHm*
1	31m 50 ug	151322.00	57490.73	2.6321	227932.38	0.6639	0.9685	0.2522	2.3672
2	31m 100 ug	222679.79	65436.37	3.4030	250779.74	0.8879	1.2953	0.2609	2.4489
3	15m 50 ug	176329.10	51012.62	3.4566	308205.8	0.5721	0.8346	0.1655	1.5534
4	15m 100 ug	233983.96	56144.75	4.1675	334719.48	0.6990	1.0198	0.1677	1.5742
5	7m 50 ug	203513.88	65071.95	3.1275	289208.04	0.7037	1.0265	0.2250	2.1117
6	7m 100 ug	306578.34	80859.36	3.7915	323239.71	0.9485	1.3836	0.2502	2.3477
7	18m 49 ug	98976.08	17815.50	5.5556	246929.86	0.4008	0.5847	0.0721	0.6771
8	18m 98 ug	148617.89	29101.77	5.1068	314426.99	0.4727	0.6895	0.0926	0.8686
9	CAHm 100 ug	187758.31	29184.23	6.4336	273898.51	0.6855	1.0000	0.1066	1.0000

Gel 2

Lane	Sample ID and protein loading	PR-A	PR-B	PR-A/PR-B	GAPDH	PR-A/GAPDH	Rel to CAHm*	PR-B/GAPDH	Rel to CAHm*
1	19m 50 ug	54310.35	25791.61	2.1057	293383.56	0.1851	0.2112	0.0879	0.5399
2	19m 100 ug	66080.61	18894.82	3.4973	329397.00	0.2006	0.2289	0.0574	0.3523
3	L1m 43.4 ug	24844.34	6236.73	3.9836	225669.52	0.1101	0.1256	0.0276	0.1697
4	L1m 86.8 ug	62514.83	13160.48	4.7502	388775.56	0.1608	0.1835	0.0339	0.2079
5	CAHm 50 ug	167522.73	42286.57	3.9616	256163.17	0.6540	0.7462	0.1651	1.0138
6	CAHm 100 ug	212758.2	44826.98	4.7462	309319.92	0.6878	0.7848	0.1449	0.8900
7	35m 50 ug	192362.87	20262.07	9.4937	268670.00	0.7160	0.8169	0.0754	0.4632
8	35m 100 ug	275702.54	56135.28	4.9114	361060.77	0.7636	0.8713	0.1555	0.9548
9	CAHm 100 ug	258827.6	48088.01	5.3824	295326.86	0.8764	1.0000	0.1628	1.0000

Gel 3

Lane	Sample ID and protein loading	PR-A	PR-B	PR-A/PR-B	GAPDH	PR-A/GAPDH	Rel to CAHm*	PR-B/GAPDH	Rel to CAHm*
1	13m 50 ug	138588.13	17531.32	7.9052	202371.14	0.6848	0.5485	0.0866	0.3290
2	13m 100 ug	243636.24	47939.12	5.0822	303254.00	0.8034	0.6435	0.1581	0.6003
3	20m 50 ug	181888.71	40031.82	4.5436	260081.50	0.6994	0.5601	0.1539	0.5845
4	20m 100 ug	288440.15	76463.53	3.7723	368654.94	0.7824	0.6267	0.2074	0.7876
5	14m 37.3 ug	36192.51	9165.84	3.9486	224500.54	0.1612	0.1291	0.0408	0.1550
6	14m 74.5 ug	58731.01	18743.23	3.1335	291120.68	0.2017	0.1616	0.0644	0.2445
7	L3m 50 ug	53401.07	22497.89	2.3736	192482.12	0.2774	0.2222	0.1169	0.4438
8	L3m 75 ug	46489.38	27357.42	1.6993	189575.87	0.2452	0.1964	0.1443	0.5480
9	CAHm 100 ug	241379.31	50913.2	4.7410	193330.00	1.2485	1.0000	0.2633	1.0000

Gel 4

Lane	Sample ID and protein loading	PR-A	PR-B	PR-A/PR-B	GAPDH	PR-A/GAPDH	Rel to CAHm*	PR-B/GAPDH	Rel to CAHm*
1	38m 50 ug	76204.00	4404.00	17.3034	20869.88	3.6514	0.4885	0.2110	0.1717
2	38m 100 ug	188288.77	21232.08	8.8681	32317.21	5.8263	0.7795	0.6570	0.5346
3	75m 50 ug	94788.50	7126.25	13.3013	31797.84	2.9810	0.3988	0.2241	0.1824
4	75m 100 ug	135326.42	8378.70	16.1512	55609.64	2.4335	0.3256	0.1507	0.1226
5	17m 50 ug	69067.85	8255.18	8.3666	33890.90	2.0379	0.2727	0.2436	0.1982
6	17m 100 ug	114499.50	14000.75	8.1781	53352.33	2.1461	0.2871	0.2624	0.2136
7	66m 50 ug	138194.42	11283.56	12.2474	28429.43	4.8610	0.6503	0.3969	0.3230
8	66m 100 ug	256576.50	58646.54	4.3750	46401.87	5.5294	0.7398	1.2639	1.0285
9	CAHm 100 ug	275512.31	45295.09	6.0826	36860.00	7.4746	1.0000	1.2288	1.0000

Gel 5

Lane	Sample ID and protein loading	PR-A	PR-B	PR-A/PR-B	GAPDH	PR-A/GAPDH	Rel to CAHm*	PR-B/GAPDH	Rel to CAHm*
1	25m 50 ug	42020.17	11239.62	3.7386	343375.94	0.1224	0.1141	0.0327	0.1890
2	25m 100 ug	62599.38	20608.48	3.0376	412278.71	0.1518	0.1415	0.0500	0.2887
3	L4m 50 ug	22535.22	2023.02	11.1394	351494.78	0.0641	0.0598	0.0058	0.0332
4	L4m 100 ug	58572.25	9978.13	5.8701	540909.57	0.1083	0.1009	0.0184	0.1065
5	51m 50 ug	132610.95	10170.44	13.0389	227403.27	0.5832	0.5436	0.0447	0.2583
6	51m 100 ug	242187.65	28964.50	8.3615	331062.09	0.7315	0.6820	0.0875	0.5053
7	94m 50 ug	256353.01	34463.55	7.4384	261105.65	0.9818	0.9152	0.1320	0.7623
8	94m 100 ug	368688.70	66980.15	5.5044	359695.94	1.0250	0.9555	0.1862	1.0754
9	CAHm 100 ug	394653.54	63703.15	6.1952	367897.58	1.0727	1.0000	0.1732	1.0000

Gel 6

Lane	Sample ID and protein loading	PR-A	PR-B	PR-A/PR-B	GAPDH	PR-A/GAPDH	Rel to CAHm*	PR-B/GAPDH	Rel to CAHm*
1	37m 50 ug	253969.38	91916.32	2.7630	43167.40	5.8834	1.5101	2.1293	6.1523
2	37m 100 ug	320987.69	106878.71	3.0033	50619.39	6.3412	1.6276	2.1114	6.1007
3	58m 50 ug	140486.92	9540.35	14.7256	36619.75	3.8364	0.9847	0.2605	0.7528
4	58m 100 ug	217332.65	14315.00	15.1822	70900.28	3.0653	0.7868	0.2019	0.5834
5	26m 50 ug	249904.08	40220.07	6.2134	66027.41	3.7849	0.9715	0.6091	1.7600
6	26m 100 ug	397735.35	106909.62	3.7203	86799.57	4.5822	1.1761	1.2317	3.5588
7	L5m 50 ug	39118.60	4768.03	8.2044	46015.51	0.8501	0.2182	0.1036	0.2994
8	L5m 100 ug	44951.40	8976.53	5.0077	66029.26	0.6808	0.1747	0.1359	0.3928
9	CAHm 100 ug	217496.57	19320.90	11.2571	55825.14	3.8960	1.0000	0.3461	1.0000

Gel 7

Lane	Sample ID and protein loading	PR-A	PR-B	PR-A/PR-B	GAPDH	PR-A/GAPDH	Rel to CAHm*	PR-B/GAPDH	Rel to CAHm*
1	74m 50 ug	253960.54	37901.46	6.7005	137862.20	1.8421	3.2333	0.2749	5.4187
2	74m 100 ug	387594.49	100329.79	3.8632	249215.07	1.5553	2.7298	0.4026	7.9349
3	95m 50 ug	147751.94	16543.21	8.9313	248128.20	0.5955	1.0452	0.0667	1.3141
4	95m 100 ug	227127.57	31171.39	7.2864	267501.75	0.8491	1.4903	0.1165	2.2967
5	52m 50 ug	117031.11	7710.97	15.1772	182281.20	0.6420	1.1269	0.0423	0.8338
6	52m 100 ug	192125.77	35484.34	5.4144	292258.00	0.6574	1.1538	0.1214	2.3931
7	84m 50 ug	43836.63	6676.00	6.5663	235064.22	0.1865	0.3273	0.0284	0.5598
8	84m 100 ug	152805.46	18050.53	8.4654	294426.06	0.5190	0.9109	0.0613	1.2084
9	CAHm 100 ug	126674.32	11280.55	11.2294	222338.13	0.5697	1.0000	0.0507	1.0000

Gel 8

Lane	Sample ID and protein loading	PR-A	PR-B	PR-A/PR-B	GAPDH	PR-A/GAPDH	Rel to CAHm*	PR-B/GAPDH	Rel to CAHm*
1	30m 50 ug	54861.77	8862.37	6.1904	71968.39	0.7623	0.3505	0.1231	0.2253
2	30m 100 ug	142899.95	42049.75	3.3984	116428.71	1.2274	0.5643	0.3612	0.6608
3	L6m 50 ug	3112.00	2573.03	1.2095	85032.33	0.0366	0.0168	0.0303	0.0554
4	L6m 100 ug	8221.11	3271.00	2.5133	91125.20	0.0902	0.0415	0.0359	0.0657
5	CAHm 100 ug	202159.65	50799.13	3.9796	92949.07	2.1750	1.0000	0.5465	1.0000
6	100m 50 ug	102148.00	14474.63	7.0570	73005.18	1.3992	0.6433	0.1983	0.3628
7	100m 100 ug	153542.58	20779.95	7.3890	104352.83	1.4714	0.6765	0.1991	0.3644
8	81m 50 ug	64467.75	11385.00	5.6625	87735.74	0.7348	0.3378	0.1298	0.2374
9	81m 100 ug	60012.49	13278.38	4.5196	89770.13	0.6685	0.3074	0.1479	0.2706

Gel 9

Lane	Sample ID and protein loading	PR-A	PR-B	PR-A/PR-B	GAPDH	PR-A/GAPDH	Rel to CAHm*	PR-B/GAPDH	Rel to CAHm*
1	86m 50 ug	250818.20	64655.43	3.8793	160419.60	1.5635	0.6672	0.4030	1.3221
2	86m 100 ug	457996.31	191781.71	2.3881	233397.02	1.9623	0.8374	0.8217	2.6954
3	29m 50 ug	75033.54	12935.79	5.8005	134879.72	0.5563	0.2374	0.0959	0.3146
4	29m 100 ug	163631.54	17152.18	9.5400	238583.67	0.6858	0.2927	0.0719	0.2358
5	L7m 50 ug	64106.77	8739.76	7.3351	149059.77	0.4301	0.1835	0.0586	0.1923
6	L7m 100 ug	121605.16	22737.58	5.3482	176209.06	0.6901	0.2945	0.1290	0.4233
7	CAHm 100 ug	303345.40	39463.25	7.6868	129452.07	2.3433	1.0000	0.3048	1.0000

Gel 10

Lane	Sample ID and protein loading	PR-A	PR-B	PR-A/PR-B	GAPDH	PR-A/GAPDH	Rel to CAHm*	PR-B/GAPDH	Rel to CAHm*
1	191m 50 ug	221813.15	49696.67	4.4633	117161.23	1.8932	1.7729	0.4242	3.6556
2	191m 100 ug	396789.78	110353.46	3.5956	208589.69	1.9023	1.7813	0.5290	4.5594
3	192m 50 ug	210004.19	32216.93	6.5184	159465.75	1.3169	1.2332	0.2020	1.7411

Key to Sample ID

Group	Sample
"45d"	31m
	CAHm
	38m
	51m
	74m
	217m
	296m
	321m
"55dA"	15m
	35m
	75m
	94m
	95m
	100m
	294m
	658m
"55dB"	7m
	13m, mng
	37m
	52m, mng
	81m
	322m
	295m, mng
	649m
"62d"	18m
	20m
	66m
	58m
	84m
	86m, mng
	258m, mng
	329m, mng
"67d"	19m
	14m
	25m
	26m, mng
	29m
	214m, mng
	263m
	310m
Labor	L1m
	L3m, mng
	L4m
	L5m
	L6m
	L7m

mng, non-gravid horn

4	192m 100 ug	335412.25	83112.42	4.0356	260547.85	1.2873	1.2055	0.3190	2.7491
5	CAHm 100 ug	240139.27	26092.83	9.2033	224872.46	1.0679	1.0000	0.1160	1.0000
6	194m 50 ug	256028.49	29866.94	8.5723	171030.61	1.4970	1.4018	0.1746	1.5050
7	194m 100 ug	382353.60	60927.82	6.2755	224663.99	1.7019	1.5937	0.2712	2.3372
8	241m 50 ug	89471.29	16118.46	5.5509	161585.53	0.5537	0.5185	0.0998	0.8597
9	241m 100 ug	104735.46	20840.24	5.0256	165306.06	0.6336	0.5933	0.1261	1.0865

Gel 11

Lane	Sample ID and protein loading	PR-A	PR-B	PR-A/PR-B	GAPDH	PR-A/GAPDH	Rel to CAHm*	PR-B/GAPDH	Rel to CAHm*
1	156m 50 ug	178551.17	34369.89	5.1950	102853.00	1.7360	0.9909	0.3342	1.1202
2	156m 100 ug	349645.82	133631.87	2.6165	103737.35	3.3705	1.9239	1.2882	4.3184
3	224m 50 ug	95048.25	13153.91	7.2259	131171.42	0.7246	0.4136	0.1003	0.3362
4	224m 100 ug	145924.96	30710.94	4.7516	184701.54	0.7901	0.4510	0.1663	0.5574
5	CAHm 100 ug	287095.27	48884.65	5.8729	163877.05	1.7519	1.0000	0.2983	1.0000
6	230m 50 ug	427781.44	116195.17	3.6816	120564.32	3.5482	2.0253	0.9638	3.2308
7	230m 100 ug	618135.48	182423.11	3.3885	138752.97	4.4549	2.5429	1.3147	4.4074
8	233m 50 ug	61809.94	16741.50	3.6920	133119.50	0.4643	0.2650	0.1258	0.4216
9	233m 100 ug	94801.91	36995.93	2.5625	195311.79	0.4854	0.2771	0.1894	0.6350

Gel 12

Lane	Sample ID and protein loading	PR-A	PR-B	PR-A/PR-B	GAPDH	PR-A/GAPDH	Rel to CAHm*	PR-B/GAPDH	Rel to CAHm*
1	197m 47.4 ug	53414.68	7095.68	7.5278	239813.95	0.2227	0.3185	0.0296	0.7271
2	197m 94.8 ug	140214.14	25713.70	5.4529	364991.15	0.3842	0.5494	0.0705	1.7312
3	225m 50 ug	134024.77	6689.75	20.0343	203535.10	0.6585	0.9417	0.0329	0.8077
4	225m 100 ug	189425.70	16910.72	11.2015	314560.14	0.6022	0.8612	0.0538	1.3211
5	CAHm 100 ug	186809.33	10872.00	17.1826	267167.07	0.6992	1.0000	0.0407	1.0000
6	232m 50 ug	344278.71	22029.71	15.6279	201711.33	1.7068	2.4410	0.1092	2.6838
7	232m 100 ug	438382.27	92977.22	4.7149	270826.80	1.6187	2.3150	0.3433	8.4364
8	219m 50 ug	42305.09	2562.88	16.5069	193667.67	0.2184	0.3124	0.0132	0.3252
9	219m 100 ug	81105.93	6002.18	13.5127	225307.14	0.3600	0.5148	0.0266	0.6546

Gel 13

Lane	Sample ID and protein loading	PR-A	PR-B	PR-A/PR-B	GAPDH	PR-A/GAPDH	Rel to CAHm*	PR-B/GAPDH	Rel to CAHm*
1	226m 50 ug	49442.07	8186.10	6.0398	215244.40	0.2297	0.2088	0.0380	0.1417
2	226m 100 ug	128161.33	21846.00	5.8666	358860.40	0.3571	0.3246	0.0609	0.2268
3	83m 50 ug	79831.02	9155.21	8.7197	242903.94	0.3287	0.2987	0.0377	0.1404
4	83m 100 ug	191863.47	25849.83	7.4222	336069.51	0.5709	0.5189	0.0769	0.2865
5	CAHm 100 ug	244003.66	59536.26	4.0984	221764.56	1.1003	1.0000	0.2685	1.0000
6	216m 50 ug	246613.85	46645.23	5.2870	88293.62	2.7931	2.5385	0.5283	1.9678
7	216m 100 ug	440709.11	116105.35	3.7958	183596.21	2.4004	2.1816	0.6324	2.3556
8	207m 50 ug	333525.47	92006.04	3.6250	57411.90	5.8093	5.2799	1.6026	5.9693
9	207m 100 ug	454904.44	148060.33	3.0724	76277.52	5.9638	5.4203	1.9411	7.2302

Westerns Jan-Mar 2010

Gel 14

Lane	Sample ID and protein loading	PR-A	PR-B	PR-A/PR-B	GAPDH	PR-A/GAPDH	Rel to CAHm*	PR-B/GAPDH	Rel to CAHm*
1	296m 50 ug	10704802.63	1936635.13	5.5275	16373387.02	0.6538	0.7688	0.1183	0.6310
2	296m 100 ug	15398093.57	3823481.22	4.0272	16921627.54	0.9100	1.0701	0.2260	1.2055
3	294m 50 ug	7320485.71	906916.14	8.0718	17304311.76	0.4230	0.4975	0.0524	0.2796
4	294m 100 ug	13437534.85	1924539.18	6.9822	17010365.68	0.7900	0.9290	0.1131	0.6036
5	CAHm 100 ug	13813479.53	3044767.65	4.5368	16244073.12	0.8504	1.0000	0.1874	1.0000
6	322m 50 ug	6320801.68	360923.55	17.5129	18141356.00	0.3484	0.4097	0.0199	0.1061
7	322m 100 ug	10660436.62	550068.17	19.3802	18370328.00	0.5803	0.6824	0.0299	0.1597
8	258m 50 ug	6713094.39	493652.69	13.5988	15721060.64	0.4270	0.5021	0.0314	0.1675
9	258m 100 ug	8797546.31	2100497.00	4.1883	15247173.51	0.5770	0.6785	0.1378	0.7350

Gel 15

Lane	Sample ID and protein loading	PR-A	PR-B	PR-A/PR-B	GAPDH	PR-A/GAPDH	Rel to CAHm*	PR-B/GAPDH	Rel to CAHm*
1	258mng 50 ug	6397442.42	1309432.14	4.8857	14620873.65	0.4376	1.5752	0.0896	1.5334
2	258mng 100 ug	9441506.14	1983688.86	4.7596	13857604.56	0.6813	2.4527	0.1431	2.4510
3	295m 50 ug	3266382.81	546385.50	5.9782	10492434.62	0.3113	1.1207	0.0521	0.8916
4	295m 100 ug	3937589.22	1306971.07	3.0128	10561267.13	0.3728	1.3422	0.1238	2.1189
5	CAHm 100 ug	2812848.23	591407.03	4.7562	10126081.65	0.2778	1.0000	0.0584	1.0000
6	295mng 50 ug	10308722.66	1167703.40	8.8282	9956826.51	1.0353	3.7272	0.1173	2.0080
7	295mng 100 ug	11797799.25	3082806.20	3.8270	10114159.07	1.1665	4.1992	0.3048	5.2188
8	329m 50 ug	2341701.83	324555.75	7.2151	12554893.80	0.1865	0.6714	0.0259	0.4426
9	329m 100 ug	2246600.50	757700.23	2.9650	12874800.40	0.1745	0.6282	0.0589	1.0077

Gel 16

Lane	Sample ID and protein loading	PR-A	PR-B	PR-A/PR-B	GAPDH	PR-A/GAPDH	Rel to CAHm*	PR-B/GAPDH	Rel to CAHm*
1	329mng 50 ug	6007280.09	1821781.00	3.2975	12006780.00	0.5003	1.4674	0.1517	1.5990
2	329mng 100 ug	8542882.79	2737447.50	3.1207	8911795.37	0.9586	2.8114	0.3072	3.2372
3	263m 50 ug	3233682.53	420320.63	7.6934	12343643.72	0.2620	0.7683	0.0341	0.3589
4	263m 100 ug	5194080.89	823814.43	6.3049	12353608.80	0.4205	1.2331	0.0667	0.7028
5	CAHm 100 ug	3312499.41	921827.44	3.5934	9714972.00	0.3410	1.0000	0.0949	1.0000
6	L3mng 50 ug	10104343.91	2401434.60	4.2076	11985429.28	0.8431	2.4725	0.2004	2.1116
7	L3mng 50 ug	14564360.35	3448355.88	4.2236	11596511.03	1.2559	3.6834	0.2974	3.1338
8	321m 50 ug	7224489.25	1201393.14	6.0134	11557557.64	0.6251	1.8333	0.1039	1.0955
9	321m 100 ug	6246522.67	1329160.71	4.6996	12791153.99	0.4883	1.4322	0.1039	1.0951

Gel 17

Lane	Sample ID and protein loading	PR-A	PR-B	PR-A/PR-B	GAPDH	PR-A/GAPDH	Rel to CAHm*	PR-B/GAPDH	Rel to CAHm*
1	13mng 50 ug	4065868.51	843094.85	4.8226	11073733.54	0.3672	1.7959	0.0761	2.6991
2	13mng 100 ug	4745297.48	1090774.70	4.3504	10077833.03	0.4709	2.3031	0.1082	3.8371
3	CAHm 100 ug	2091520.43	288564.77	7.2480	10229944.67	0.2045	1.0000	0.0282	1.0000
4	52mng 50 ug	9034312.13	952820.41	9.4817	8197848.11	1.1020	5.3902	0.1162	4.1204
5	52mng 50 ug	8730028.68	935355.18	9.3334	8522425.25	1.0244	5.0103	0.1098	3.8908
6	310m 50 ug	1310099.74	264679.86	4.9498	11895227.75	0.1101	0.5387	0.0223	0.7888
7	310m 100 ug	1608930.79	508294.88	3.1653	13735884.69	0.1171	0.5729	0.0370	1.3119

Gel 18

Lane	Sample ID and protein loading	PR-A	PR-B	PR-A/PR-B	GAPDH	PR-A/GAPDH	Rel to CAHm*	PR-B/GAPDH	Rel to CAHm*
1	86mng 50 ug	18162290.24	4576726.88	3.9684	15340634.50	1.1839	2.8883	0.2983	3.3348
2	86mng 100 ug	23460977.61	7760643.26	3.0231	13343412.14	1.7582	4.2893	0.5816	6.5012
3	26mng 50 ug	31292198.61	5710334.04	5.4799	11977310.08	2.6126	6.3736	0.4768	5.3292
4	26mng 50 ug	32674005.83	6480782.97	5.0417	15140893.00	2.1580	5.2645	0.4280	4.7845
5	CAHm 100 ug	4891104.11	1067469.21	4.5820	11932094.38	0.4099	1.0000	0.0895	1.0000

Gel 19

Lane	Sample ID and protein loading	PR-A	PR-B	PR-A/PR-B	GAPDH	PR-A/GAPDH	Rel to CAHm*	PR-B/GAPDH	Rel to CAHm*
1	214m 50 ug	5610940.05	1873417.00	2.9950	25182533.86	0.2228	0.4013	0.0744	0.5451
2	214m 100 ug	8035079.81	2990928.56	2.6865	26263877.60	0.3059	0.5510	0.1139	0.8344
3	214mng 50 ug	15893590.45	2951085.80	5.3857	18087065.37	0.8787	1.5826	0.1632	1.1954
4	649m 50 ug	16266469.45	2708552.58	6.0056	21538109.68	0.7552	1.3602	0.1258	0.9214
5	649m 100 ug	16729651.07	5413658.96	3.0903	31200419.29	0.5362	0.9657	0.1735	1.2713
6	CAHm 100 ug	10116097.65	2486609.23	4.0682	18218720.47	0.5553	1.0000	0.1365	1.0000
7	217m 50 ug	10733615.82	2271682.50	4.7250	27783443.03	0.3863	0.6958	0.0818	0.5991
8	217m 100 ug	20193729.00	4811666.36	4.1968	25948107.74	0.7782	1.4016	0.1854	1.3586
9	658m 50 ug	21294070.74	4827443.03	4.4110	19779742.56	1.0766	1.9388	0.2441	1.7882
10	658m 100 ug	27661880.04	8581949.65	3.2233	26027484.93	1.0628	1.9141	0.3297	2.4158

*, sample used as calibrator
