# Supplementary material for: Progesterone Receptor Expression Declines in the Guinea Pig Uterus during Functional Progesterone Withdrawal and in Response to Prostaglandins
Source: PLoS One. 2014 Aug 26;9(8):e105253. doi: 10.1371/journal.pone.0105253 (PMC4144885; doi:10.1371/journal.pone.0105253)
Supplement: Table S2 — Densitometric evaluation of estrogen receptor (ESR1) immunoblots presented in Figure S4. (PDF) [file pone.0105253.s009.pdf]

Table S2

Guinea Pig Uterine Estrogen Receptor Protein (ESR1) Levels During Pregnancy

(**Arbitrary Densitometric Units)**

Gel 1

Lane	Sample ID[§] and protein loading	**ESR1**	**GAPDH**	**ESR1/GAPDH**	**Rel to T47D***
1	31m 60 µg	2813575.44	49785710.00	0.0565	0.3911
2	31m 60 µg	2954729.70	44245540.93	0.0668	0.4621
3	15m 60 µg	2265224.38	72454050.10	0.0313	0.2164
4	15m 60 µg	2378301.45	66258636.68	0.0359	0.2484
5	T47D 20 µg	4546421.00	31463131.68	0.1445	1.0000
6	7m 60 µg	3162714.50	68242181.88	0.0463	0.3207
7	7m 60 µg	3141394.17	58147880.59	0.0540	0.3739
8	18m 60 µg	1802218.33	88921226.97	0.0203	0.1403
9	18m 60 µg	1780962.05	88425422.83	0.0201	0.1394

Gel 2

Lane	Sample ID[§] and protein loading	**ESR1**	**GAPDH**	**ESR1/GAPDH**	**Rel to T47D***
1	19m 60 µg	9798104.55	42270564.65	0.2318	0.4371
2	L1m 60 µg	4834981.83	53280969.31	0.0907	0.1711
3	L1m 60 µg	5578929.27	54285739.00	0.1028	0.1938
4	T47D 20 µg	12770042.90	24083100.26	0.5302	1.0000
5	CAHm 60 µg	9072497.51	47814387.77	0.1897	0.3578
6	CAHm 60 µg	7000007.91	45031780.75	0.1554	0.2932
7	35m 60 µg	9866401.71	56595631.76	0.1743	0.3288
8	35m 60 µg	9770673.50	75884707.48	0.1288	0.2428

Gel 3

Lane	Sample ID[§] and protein loading	**ESR1**	**GAPDH**	**ESR1/GAPDH**	**Rel to T47D***
1	13m 60 µg	17527265.12	9584863.88	1.8286	0.6387
2	13m 60 µg	17687270.04	9333413.58	1.8950	0.6619
3	13mng 60 µg	14496152.55	6826386.96	2.1235	0.7417
4	13mng 60 µg	14969824.55	7137152.81	2.0975	0.7326
5	T47D 20 µg	15215352.76	5314151.72	2.8632	1.0000
6	20m 60 µg	17153065.91	10150930.73	1.6898	0.5902
7	20m 60 µg	15492563.23	10328106.13	1.5000	0.5239
8	14m 60 µg	13425258.11	11165257.72	1.2024	0.4200
9	14m 60 µg	13865852.00	11976583.65	1.1577	0.4044

Gel 4

Lane	Sample ID[§] and protein loading	**ESR1**	**GAPDH**	**ESR1/GAPDH**	**Rel to T47D***
1	L3m 60 µg	8341065.21	9882429.03	0.8440	0.2704
2	L3m 60 µg	8590702.18	10148761.75	0.8465	0.2712
3	L3mng 60 µg	20829713.43	9611818.77	2.1671	0.6942
4	L3mng 60 µg	20063982.98	11112873.21	1.8055	0.5783
5	T47D 20 µg	17690851.00	5666874.40	3.1218	1.0000
6	38m 60 µg	20771882.50	9607968.28	2.1619	0.6925
7	38m 60 µg	20077036.92	10407540.88	1.9291	0.6179
8	75m 60 µg	22039057.00	12003254.61	1.8361	0.5882
9	75m 60 µg	21908512.73	13657014.24	1.6042	0.5139

Gel 5

Lane	Sample ID[§] and protein loading	**ESR1**	**GAPDH**	**ESR1/GAPDH**	**Rel to T47D***
1	37m 60 µg	7129955.57	14020901.08	0.5085	0.5404
2	37m 60 µg	7588054.76	14434414.37	0.5257	0.5586
3	66m 60 µg	7719322.50	13636536.36	0.5661	0.6016
4	66m 60 µg	7380379.72	13995561.10	0.5273	0.5604
5	T47D 20 µg	7152663.67	7600900.43	0.9410	1.0000
6	25m 60 µg	6696166.07	14179924.90	0.4722	0.5018
7	25m 60 µg	7688479.25	12382899.35	0.6209	0.6598
8	L4m 60 µg	24112107.50	13423744.09	1.7962	1.9088
9	L4m 60 µg	22891828.67	14046587.23	1.6297	1.7318

Gel 6

Lane	Sample ID[§] and protein loading	**ESR1**	**GAPDH**	**ESR1/GAPDH**	**Rel to T47D***
1	51m 60 µg	3806065.13	15413353.75	0.2469	0.4184
2	51m 60 µg	2951590.55	14332255.46	0.2059	0.3490
3	94m 60 µg	2180562.71	15225113.92	0.1432	0.2427
4	94m 60 µg	2154526.57	15214687.41	0.1416	0.2400
5	T47D 20 µg	4909981.08	8320060.75	0.5901	1.0000
6	52m 60 µg	6101338.56	15261776.26	0.3998	0.6774
7	52m 60 µg	6225957.64	17053121.64	0.3651	0.6187
8	52mng 60 µg	10906887.33	15541481.06	0.7018	1.1892
9	52mng 60 µg	11675339.4	19202143.08	0.6080	1.0303

Gel 7

Lane	Sample ID[§] and protein loading	**ESR1**	**GAPDH**	**ESR1/GAPDH**	**Rel to T47D***
1	58m 60 µg	7805740.33	14705065.57	0.5308	0.3922
2	58m 60 µg	7342023.00	10363124.69	0.7085	0.5234
3	26m 60 µg	6410287.39	14089882.28	0.4550	0.3361
4	26m 60 µg	6294653.64	10186563.77	0.6179	0.4565
5	T47D 20 µg	7625886.00	5633967.25	1.3536	1.0000
6	26mng 60 µg	12409900.71	7126445.24	1.7414	1.2865
7	26mng 60 µg	11813445.57	7157250.89	1.6506	1.2194

8	L5m 60 µg	7580237.87	11003142.76	0.6889	0.5090
9	L5m 60 µg	6881063.59	12010517.46	0.5729	0.4233

Gel 8

Lane	Sample ID§ and protein loading	ESR1	GAPDH	ESR1/GAPDH	Rel to T47D*
1	74m 60 µg	4648525.86	8230686.60	0.5648	0.4295
2	74m 60 µg	4418417.00	7322931.73	0.6034	0.4589
3	95m 60 µg	4510813.58	7666127.36	0.5884	0.4475
4	95m 60 µg	4769647.00	8229168.31	0.5796	0.4408
5	T47D 20 µg	7828945.21	5953921.86	1.3149	1.0000
6	81m 60 µg	5546212.25	10070461.30	0.5507	0.4188
7	81m 60 µg	5606342.19	8755614.65	0.6403	0.4870
8	84m 60 µg	8005492.23	9928900.26	0.8063	0.6132
9	84m 60 µg	8781563.83	11490525.59	0.7642	0.5812

Gel 9

Lane	Sample ID§ and protein loading	ESR1	GAPDH	ESR1/GAPDH	Rel to T47D*
1	29myo 50 µg	5460741.10	31274237.64	0.1746	0.5811
2	29myo 50 µg	6114347.59	21717955.28	0.2815	0.9370
3	L6myo 50 µg	7491156.00	15510163.37	0.4830	1.6074
4	L6myo 50 µg	6814371.84	15894779.87	0.4287	1.4268
5	T47D 20 µg	2915829.00	9704152.86	0.3005	1.0000
6	217myo 50 µg	7232612.60	9456376.39	0.7648	2.5455
7	217myo 50 µg	7746274.00	11175143.13	0.6932	2.3069
8	100myo 50 µg	6124180.92	13907202.98	0.4404	1.4656
9	100myo 50 µg	4373459.89	15371226.86	0.2845	0.9469

Gel 10

Lane	Sample ID§ and protein loading	ESR1	GAPDH	ESR1/GAPDH	Rel to T47D*
1	322m 50 µg	4739486.59	10873426.97	0.4359	1.0424
2	322m 50 µg	6265092.19	9261899.95	0.6764	1.6177
3	86m 50 µg	3943028.00	16969744.60	0.2324	0.5557
4	86m 50 µg	3880215.28	21455094.74	0.1809	0.4325
5	T47D 20 µg	5310375.56	12699502.65	0.4182	1.0000
6	86mng 50 µg	8682527.15	12592651.42	0.6895	1.6489
7	86mng 50 µg	8000749.60	13041255.45	0.6135	1.4671
8	214m 50 µg	8359927.00	24620606.43	0.3396	0.8120
9	214m 50 µg	4884126.59	28255194.79	0.1729	0.4134

Gel 11

Lane	Sample ID§ and protein loading	ESR1	GAPDH	ESR1/GAPDH	Rel to T47D*
1	214mng 50 µg	10847905.96	8937988.43	1.2137	1.0282
2	214mng 50 µg	12371951.43	7337958.46	1.6860	1.4283
3	L7m 50 µg	6815134.81	7834188.60	0.8699	0.7370
4	L7m 50 µg	6452510.96	6885321.76	0.9371	0.7939
5	T47D 20 µg	5549948.67	4701644.52	1.1804	1.0000
6	296m 50 µg	5128339.91	3833028.71	1.3379	1.1334
7	296m 50 µg	5209808.31	3729376.08	1.3970	1.1834
8	294m 50 µg	16718958.57	4169734.42	4.0096	3.3967
9	294m 50 µg	14919943.75	4238392.41	3.5202	2.9821

Gel 12

Lane	Sample ID§ and protein loading	ESR1	GAPDH	ESR1/GAPDH	Rel to T47D*
1	295m 50 µg	4810918.65	10979190.45	0.4382	0.2562
2	295m 50 µg	4661696.45	9403229.43	0.4958	0.2898
3	295mng 50 µg	10151980.85	8908085.94	1.1396	0.6662
4	295mng 50 µg	11001518.88	8940662.52	1.2305	0.7194
5	T47D 20 µg	10265604.32	6001420.00	1.7105	1.0000
6	258m 50 µg	6113928.79	9031714.56	0.6769	0.3957
7	258m 50 µg	6620321.97	9155152.62	0.7231	0.4227
8	258mng 50 µg	10869563.63	9153283.72	1.1875	0.6942
9	258mng 50 µg	8643259.54	8144580.47	1.0612	0.6204

Gel 13

Lane	Sample ID§ and protein loading	ESR1	GAPDH	ESR1/GAPDH	Rel to T47D*
1	263m 50 µg	24769819.89	44761694.8	0.5534	0.5866
2	263m 50 µg	26783932.61	40291032.95	0.6648	0.7047
3	321m 50 µg	29665297.11	6519077.18	4.5505	4.8240
4	321m 50 µg	29021103.61	6414367.45	4.5244	4.7963
5	T47D 20 µg	25041095.11	26545903.57	0.9433	1.0000
6	658m 50 µg	46625463.71	27810748	1.6765	1.7773
7	658m 50 µg	43506917.19	28006247.19	1.5535	1.6468
8	649m 50 µg	29222970.3	29516607.88	0.9901	1.0495
9	649m 50 µg	2770921.75	3540214.67	0.7827	0.8297

Gel 14

Lane	Sample ID§ and protein loading	ESR1	GAPDH	ESR1/GAPDH	Rel to T47D*
1	329m 50 µg	37388121.00	10138325.66	3.6878	1.4738
2	329m 50 µg	36631683.43	8418990.61	4.3511	1.7389
3	329mng 50 µg	40948057.00	9122734.33	4.4886	1.7938
4	310m 50 µg	45391312.87	16628775.66	2.7297	1.0909
5	T47D 20 µg	33873419.75	13537234.59	2.5022	1.0000
6	310m 50 µg	41954106.62	18633127.10	2.2516	0.8998

*, T47D cell extract used as calibrator

§, see Supplementary Table S1 for Key to Sample ID
