# Supplementary material for: Progesterone Receptor Expression Declines in the Guinea Pig Uterus during Functional Progesterone Withdrawal and in Response to Prostaglandins
Source: PLoS One. 2014 Aug 26;9(8):e105253. doi: 10.1371/journal.pone.0105253 (PMC4144885; doi:10.1371/journal.pone.0105253)
Supplement: Table S3 — Densitometric evaluation of progesterone receptor and ESR1 immunoblots presented in Figure S5 and Figure S6, respectively (Sulprostone study). (PDF) [file pone.0105253.s010.pdf]

Table S3

Effect of Sulprostone on Guinea Pig Uterine Progesterone Receptor and Estrogen Receptor Protein Levels

Key to Sample ID	
**Vehicle**	**Sulprostone**
452m	646m, mng
454m, mng	403m
456m	441m*
416m	442m
459m, mng	443m
648m	641m
678m	677m
685m, mng	686m
	**Fluprostenol**#
	426m
	370m

mng, non-gravid horn (not evaluated)

#, Fluprostenol treatment was not evaluated

Progesterone Receptor (PRA and PRB, arbitrary densitometric units)

Gel 1

Lane	Sample ID and protein loading	PR-A	PR-B	PR-A/B	GAPDH	PR-A/GAPDH	Rel to 441m*	PR-B/GAPDH	Rel to 441m*
1	452m 50µg	6375565	1343273.94	4.7463	49777605.68	0.1281	1.2538	0.0270	1.0293
2	646m 50µg	4399335.39	786240.83	5.5954	43162812	0.1019	0.9978	0.0182	0.6948
3	646mng 50µg	2960456.71	183471.07	16.1358	50580321.94	0.0585	0.5730	0.0036	0.1383
4	441m 50µg	4830324.11	1239750.94	3.8962	47285408.45	0.1022	1.0000	0.0262	1.0000
5	403m 50µg	6123354	690911.47	8.8627	43982091.06	0.1392	1.3629	0.0157	0.5992
6	454m 50µg	4711611.14	593982.69	7.9322	44170920.59	0.1067	1.0442	0.0134	0.5129
7	454mng 50µg	15587638.77	1401901.5	11.1189	44826385.28	0.3477	3.4041	0.0313	1.1928
8	456m 50µg	4084540.23	596847.88	6.8435	42204464.51	0.0968	0.9474	0.0141	0.5394
9	442m 50µg	3140286.33	584208	5.3753	39884244.66	0.0787	0.7708	0.0146	0.5587

Gel 2

Lane	Sample ID and protein loading	PR-A	PR-B	PR-A/B	GAPDH	PR-A/GAPDH	Rel to 441m*	PR-B/GAPDH	Rel to 441m*
1	416m 50µg	7970483.75	1289853.6	6.1794	45804916.37	0.1740	1.8557	0.0282	1.0880
2	443m 50µg	4850502.14	1209442.88	4.0105	44166703.53	0.1098	1.1712	0.0274	1.0580
3	641m 50µg	2725008.05	579509.9	4.7023	47744801.39	0.0571	0.6087	0.0121	0.4689
4	441m 50µg	3814370.12	1052881.94	3.6228	40678133.84	0.0938	1.0000	0.0259	1.0000
5	459m 50µg	9231962.23	1074567.6	8.5913	47605250.48	0.1939	2.0681	0.0226	0.8721
6	459mng 50µg	29347581.88	3866837.59	7.5896	49814208.54	0.5891	6.2829	0.0776	2.9991
7	677m 50µg	3708217.38	621750	5.9642	40331928.02	0.0919	0.9805	0.0154	0.5956
8	648m 50µg	4955898	782801	6.3310	44356495.3	0.1117	1.1915	0.0176	0.6818
9	678m 50µg	4462711.44	677279.4	6.5892	41191270.63	0.1083	1.1554	0.0164	0.6352

Gel 3

Lane	Sample ID and protein loading	PR-A	PR-B	PR-A/B	GAPDH	PR-A/GAPDH	Rel to 441m*	PR-B/GAPDH	Rel to 441m*
1	686m 50µg	2409653.94	281462.76	8.5612	98999861.45	0.0243	0.3236	0.0028	0.2763
2	370m 50µg	43606812.83	9294774	4.6915	105289890	0.4142	5.5065	0.0883	8.5806
3	426m 50µg	12903152.08	1068338.5	12.0778	109964991	0.1173	1.5601	0.0097	0.9443
4	441m 50µg	8454147.05	1156416.55	7.3106	112403613.9	0.0752	1.0000	0.0103	1.0000
5	685m 50µg	16490085.76	1955933.65	8.4308	113266907.6	0.1456	1.9357	0.0173	1.6785
6	685mng 50µg	12201709.32	662692.25	18.4123	104972872.3	0.1162	1.5454	0.0063	0.6136

*, sample used as calibrator

Estrogen Receptor (ESR1, arbitrary densitometric units)

Gel 1

Lane	Sample ID and protein loading	ESR1	GAPDH	ESR1/GAPDH	Rel to 443m*
1	452m 40µg	10692931.80	668152.03	16.0037	32.5525
2	452m 40µg	10802321.33	637571.35	16.9429	34.4629
3	646m 40µg	13513578.24	11392419.42	1.1862	2.4128
4	646m 40µg	12193784.09	11763062.00	1.0366	2.1085
5	443m 40µg	10536540.00	21431916.28	0.4916	1.0000
6	646mng 40µg	23676224.17	464791.25	50.9395	103.6138
7	646mng 40µg	22519067.80	519216.04	43.3713	88.2196
8	403m 40µg	23154253.85	556864.25	41.5797	84.5755
9	403m 40µg	20505076.35	458451.91	44.7268	90.9768

Gel 2

Lane	Sample ID and protein loading	ESR1	GAPDH	ESR1/GAPDH	Rel to 443m*
1	454m 40µg	14403079.32	3777675.33	3.8127	8.2953
2	454m 40µg	14166888.60	2992267.20	4.7345	10.3009
3	454mng 40µg	13342171.93	19686608.47	0.6777	1.4745
4	454mng 40µg	16667853.38	18000169.44	0.9260	2.0147
5	443m 40µg	11525087.33	25075310.57	0.4596	1.0000
6	441m 40µg	7070268.00	34400147.00	0.2055	0.4472
7	441m 40µg	7390506.41	33157903.15	0.2229	0.4849
8	456m 40µg	27223534.79	735934.32	36.9918	80.4836
9	456m 40µg	24118392.59	570704.06	42.2608	91.9474

Gel 3

Lane	Sample ID and protein loading	ESR1	GAPDH	ESR1/GAPDH	Rel to 443m*
1	442m 40µg	29501883.87	3410397.41	8.6506	16.6642
2	442m 40µg	28136345.93	2536590.03	11.0922	21.3676
3	416m 40µg	11737221.25	6483680.03	1.8103	3.4872
4	416m 40µg	10854423.47	5773839.56	1.8799	3.6214
5	443m 40µg	14648233.74	28217878.67	0.5191	1.0000
6	443m 40µg	14640270.94	30236973.75	0.4842	0.9327
7	443m 40µg	15160125.77	31080733.34	0.4878	0.9396
8	641m 40µg	13464116.00	14577951.48	0.9236	1.7792
9	641m 40µg	13091619.00	13149944.06	0.9956	1.9178

Gel 4

Lane	Sample ID and protein loading	ESR1	GAPDH	ESR1/GAPDH	Rel to 443m*
1	459m 40µg	11022707.33	4012905.18	2.7468	4.9350
2	459m 40µg	11548055.35	3241401.96	3.5627	6.4008
3	459mng 40µg	12436321.59	48691905.85	0.2554	0.4589
4	459mng 40µg	14873003.14	44325031.18	0.3355	0.6028
5	443m 40µg	13122747.06	23576530.81	0.5566	1.0000
6	677m 40µg	15617609.11	10550559.48	1.4803	2.6595
7	677m 40µg	16236841.73	11603806.77	1.3993	2.5139
8	648m 40µg	20191794.68	2534199.92	7.9677	14.3149
9	648m 40µg	15743552.25	1834383.15	8.5825	15.4194

Gel 5

Lane	Sample ID and protein loading	ESR1	GAPDH	ESR1/GAPDH	Rel to 443m*
1	678m 40µg	12730729.00	4466505.10	2.8503	8.8283
2	678m 40µg	14517292.13	4024841.13	3.6069	11.1720
3	686m 40µg	30450312.23	3276763.79	9.2928	28.7832
4	686m 40µg	28481768.00	3197389.73	8.9078	27.5907
5	443m 40µg	11847533.21	36696112.36	0.3229	1.0000
6	685m 40µg	12420338.28	12618439.57	0.9843	3.0487
7	685m 40µg	12084419.60	12279422.77	0.9841	3.0482
8	685mng 40µg	26393105.71	1595841.84	16.5387	51.2263
9	685mng 40µg	21471935.76	1954023.70	10.9886	34.0356

Gel 6

Lane	Sample ID and protein loading	ESR1	GAPDH	ESR1/GAPDH	Rel to 443m*
1	370m 40µg	12864952.50	44270344.00	0.2906	0.3208
2	370m 40µg	13595537.67	45532124.00	0.2986	0.3296
3	426m 40µg	19561564.24	35010490.00	0.5587	0.6167
4	426m 40µg	21141779.94	39786163.00	0.5314	0.5865
5	443m 40µg	20563424.71	22696935.00	0.9060	1.0000

*, sample used as calibrator
