# Supplementary material for: Progesterone Receptor Expression Declines in the Guinea Pig Uterus during Functional Progesterone Withdrawal and in Response to Prostaglandins
Source: PLoS One. 2014 Aug 26;9(8):e105253. doi: 10.1371/journal.pone.0105253 (PMC4144885; doi:10.1371/journal.pone.0105253)
Supplement: Table S4 — Densitometric evaluation of progesterone receptor and ESR1 immunoblots presented in Figure S7 (Piroxicam study). (PDF) [file pone.0105253.s011.pdf]

Table S4

Effect of Piroxicam on Guinea Pig Uterine Progesterone Receptor and Estrogen Receptor Protein Levels

Key to Sample ID

Vehicle	Piroxicam
695m*, mng	734m
738m	795m
761m	796m
763m	768m
808m	799m
863m	857m, mng
865m	822m
	814m, mng

mng, non-gravid horn

Progesterone Receptor (PRA and PRB, arbitrary densitometric units)

Gel 1

Lane	Sample ID and protein loading	PR-A	PR-B	PR-A/B	GAPDH	PR-A/GAPDH	Rel to 695m*	PR-B/GAPDH	el to 695m*
1	734m 50 ug	7522755.04	9044939.45	0.8317	109028000.10	0.0690	0.9966	0.0830	1.0861
2	734m 75 ug	12050962.65	17068676.37	0.7060	128057712.88	0.0941	1.3592	0.1333	1.7450
3	795m 50 ug	11855205.48	19056241.91	0.6221	130190771.21	0.0911	1.3152	0.1464	1.9163
4	795m 100 ug	9829239.42	20345439.55	0.4831	140643473.59	0.0699	1.0094	0.1447	1.8939
5	695m 100 ug	9758410.89	10765738.7	0.9064	140944871.31	0.0692	1.0000	0.0764	1.0000
6	695mng 75 ug	24498140.5	13904241.39	1.7619	101781332.25	0.2407	3.4764	0.1366	1.7885
7	814m 50 ug	12463607	6806085.12	1.8312	104966842.23	0.1187	1.7150	0.0648	0.8489
8	814m 100 ug	16093782.9	8548478.6	1.8826	115178915.43	0.1397	2.0182	0.0742	0.9717
9	814mng 50 ug	23626840.69	7849852.8	3.0098	65882296.39	0.3586	5.1797	0.1191	1.5599

Gel 2

Lane	Sample ID and protein loading	PR-A	PR-B	PR-A/B	GAPDH	PR-A/GAPDH	Rel to 695m*	PR-B/GAPDH	el to 695m*
1	796m 50 ug	6186426.29	10756248.49	0.5751	60174954.88	0.1028	1.3295	0.1787	1.6210
2	796m 100 ug	7657501	19080076.27	0.4013	84974350.35	0.0901	1.1654	0.2245	2.0362
3	738m 50 ug	5083154.21	11083974.01	0.4586	58900773.53	0.0863	1.1160	0.1882	1.7065
4	738m 75 ug	7986176.14	14340155.37	0.5569	66463896.55	0.1202	1.5539	0.2158	1.9566
5	695m 100 ug	5927388.2	8452716	0.7012	76652157.46	0.0773	1.0000	0.1103	1.0000
6	768m 50 ug	8871388.76	12244426.93	0.7245	63809495.22	0.1390	1.7979	0.1919	1.7401
7	768m 100 ug	11448457.84	14597983.33	0.7842	75027610.66	0.1526	1.9733	0.1946	1.7644
8	761m 50 ug	6927705.8	13774506.88	0.5029	89489020.48	0.0774	1.0011	0.1539	1.3958
9	761m 75 ug	6637777.21	12405134.54	0.5351	107121560.1	0.0620	0.8013	0.1158	1.0502

Gel 3

Lane	Sample ID and protein loading	PR-A	PR-B	PR-A/B	GAPDH	PR-A/GAPDH	Rel to 695m*	PR-B/GAPDH	el to 695m*
1	763m 50 ug	7142876.23	7124209.11	1.0026	67674251.14	0.1055	2.0004	0.1053	3.9329
2	763m 75 ug	8513478.84	7887881.03	1.0793	65839263.41	0.1293	2.4507	0.1198	4.4758
3	799m 50 ug	10049102.07	5806738.05	1.7306	60418812.78	0.1663	3.1522	0.0961	3.5905
4	799m 75 ug	9034887.64	5885672.15	1.5351	71959099.06	0.1256	2.3796	0.0818	3.0557
5	695m 100 ug	4053368.03	2056289.29	1.9712	76821032.00	0.0528	1.0000	0.0268	1.0000
6	808m 50 ug	3453969.93	3440446.91	1.0039	65660041.90	0.0526	0.9970	0.0524	1.9575
7	808m 75 ug	4664238.71	5929009.69	0.7867	74899369.74	0.0623	1.1802	0.0792	2.9573
8	822m 50 ug	3163425	3632287.14	0.8709	68559633.20	0.0461	0.8745	0.0530	1.9793
9	822m 100 ug	6314042.45	3710042.92	1.7019	92237491.84	0.0685	1.2974	0.0402	1.5027

Gel 4

Lane	Sample ID and protein loading	PR-A	PR-B	PR-A/B	GAPDH	PR-A/GAPDH	Rel to 695m*	PR-B/GAPDH	el to 695m*
1	857m 50 ug	8329619.8	7077623.41	1.1769	81940191.18	0.1017	1.6640	0.0864	1.4448
2	857m 100 ug	6865732.17	9344759.51	0.7347	108659144.13	0.0632	1.0343	0.0860	1.4385
3	857mng 50 ug	3458825.75	6491986.28	0.5328	69685468.38	0.0496	0.8125	0.0932	1.5583
4	857mng 75 ug	5849951.02	7486470.18	0.7814	84461848.89	0.0693	1.1337	0.0886	1.4826
5	695m 100 ug	7032309.65	6881750.43	1.0219	115110824.03	0.0611	1.0000	0.0598	1.0000
6	863m 50 ug	4808891	7579454.5	0.6345	87126363.19	0.0552	0.9035	0.0870	1.4551
7	863m 100 ug	9539550.22	9412707.05	1.0135	121217360.56	0.0787	1.2882	0.0777	1.2989
8	865m 50 ug	4160794.51	7785861.8	0.5344	94837117.41	0.0439	0.7182	0.0821	1.3732
9	865m 100 ug	7761297	6811969.69	1.1394	136636356.70	0.0568	0.9298	0.0499	0.8339

*, sample used as calibrator

Estrogen Receptor (ESR1, arbitrary densitometric units)

Gel 1

Lane	Sample ID and protein loading	ESR1	GAPDH	ESR1/GAPDH	Rel to MCF-7*
1	796m 25µg	7390732.75	121336193.62	0.0609	0.0346
2	796m 25µg	7791337.67	119594737.24	0.0651	0.0370
3	738m 25µg	7140513.94	106916419.98	0.0668	0.0379
4	738m 25µg	7857750.34	97552803.37	0.0805	0.0457
5	MCF-7 10µg	16359478.48	63305049.78	0.2584	0.1467
6	695m 25µg	8607812.19	100847157.45	0.0854	0.0484
7	695m 25µg	6659602.28	101599797.82	0.0655	0.0372
8	814m 25µg	4870565.46	96016249.70	0.0507	0.0288
9	814m 25µg	5913318.04	116115103.37	0.0509	0.0289

Gel 2

Lane	Sample ID and protein loading	ESR1	GAPDH	ESR1/GAPDH	Rel to MCF-7*
1	734m 25µg	12747404.71	72729429.03	0.1753	0.2419
2	734m 25µg	14566802.57	71982802.94	0.2024	0.2793
3	865m 25µg	10423503.50	87754275.99	0.1188	0.1639
4	865m 25µg	10483470.00	91642386.75	0.1144	0.1579
5	MCF-7 10µg	10313898.22	54137027.27	0.1905	0.2630
6	795m 25µg	2128556.60	74927042.60	0.0284	0.0392
7	795m 25µg	1520347.49	77824725.51	0.0195	0.0270
8	761m 25µg	5566200.60	91376353.71	0.0609	0.0841
9	761m 25µg	6346252.84	121075480.42	0.0524	0.0723

Gel 3

Lane	Sample ID and protein loading	ESR1	GAPDH	ESR1/GAPDH	Rel to MCF-7*
1	768m 25µg	13169309.37	83988965.10	0.1568	0.1562
2	768m 25µg	12921490.96	79287104.00	0.1630	0.1623
3	763m 25µg	2696328.78	82335385.22	0.0327	0.0326
4	763m 25µg	2593776.19	86928202.50	0.0298	0.0297
5	MCF-7 10µg	11840984.64	61599990.61	0.1922	0.1915
6	799m 25µg	3662505.40	97561931.70	0.0375	0.0374
7	799m 25µg	3293117.99	85480994.60	0.0385	0.0384
8	808m 25µg	3475556.19	102264821.93	0.0340	0.0339
9	808m 25µg	3203779.17	139946449.00	0.02	0.02

Gel 4

Lane	Sample ID and protein loading	ESR1	GAPDH	ESR1/GAPDH	Rel to MCF-7*
1	857m 25µg	4690397.00	122054851.00	0.0384	0.0492
2	857m 25µg	6419097.38	111176354.69	0.0577	0.0739
3	MCF-7 10µg	13133880.14	68426013.28	0.1919	0.2456
4	MCF-7 10µg (not used as calibrator)				
5	863m 25µg	3039162.43	120722993.62	0.0252	0.0322
6	863m (assay failure)				
7	822m (assay failure)				
8	822m 25µg	3743355.44	92302077.99	0.0406	0.0519

*, MCF-7 cell extract used as calibrator
